# CrebH protects against liver injury associated with colonic inflammation via modulation of exosomal miRNA

**DOI:** 10.1186/s13578-023-01065-9

**Published:** 2023-06-27

**Authors:** Sang-Hee Lee, Sung-Je Moon, Seung Hee Woo, Gwangsook Ahn, Won Kon Kim, Chul-Ho Lee, Jung Hwan Hwang

**Affiliations:** 1 Laboratory Animal Resource Center, Korea Research Institute of Bioscience and Biotechnology (KRIBB), 125 Gwahak-ro, Yuseoung-gu, Daejeon, 34141 Korea; 2 Department of Biology, Daejeon University, 62 Daehak-ro, Dong-gu, Daejeon, 34520 Korea; 3 KRIBB School of Bioscience, University of Science and Technology (UST), 125 Gwahak-ro, Yuseong-gu, Daejeon, 34141 Korea; 4 Department of Biology and Microbiology, Changwon National University, 20 Chanwondaehak-ro, Uichan-gu, Chanwon-si, Gyeonsangnam-do 51140 Korea; 5 Metabolic Regulation Research Center, KRIBB, 125 Gwahak-ro, Yuseoung-gu, Daejeon, 34141 Korea

**Keywords:** CrebH, Exosomes, Inflammatory bowel disease, Liver damage, Primary sclerosing cholangitis

## Abstract

**Background:**

Hepatic liver disease, including primary sclerosing cholangitis (PSC), is a serious extraintestinal manifestations of colonic inflammation. Cyclic adenosine monophosphate (cAMP)-responsive element-binding protein H (CrebH) is a transcription factor expressed mostly in the liver and small intestine. However, CrebH’s roles in the gut–liver axis remain unknown.

**Methods:**

Inflammatory bowel disease (IBD) and PSC disease models were established in wild-type and *CrebH*^−/−^ mice treated with dextran sulfate sodium, dinitrobenzene sulfonic acid, and diethoxycarbonyl dihydrocollidine diet, respectively. RNA sequencing were conducted to investigate differential gene expression. Exosomes were isolated from plasma and culture media. miRNA expression profiling was performed using the NanoString nCounter Mouse miRNA Panel. Effects of miR-29a-3p on adhesion molecule expression were investigated in bEnd.3 brain endothelial cells.

**Results:**

*CrebH*^−/−^ mice exhibited accelerated liver injury without substantial differences in the gut after administration of dextran sulfate sodium (DSS), and had similar features to PSC, including enlarged bile ducts, enhanced inflammation, and aberrant MAdCAM-1 expression. Furthermore, RNA-sequencing analysis showed that differentially expressed genes in the liver of *CrebH*^−/−^ mice after DSS overlapped significantly with genes changed in PSC-liver. Analysis of plasma exosome miRNA isolated from WT and *CrebH*^−/−^ mice indicates that CrebH can contribute to the exosomal miRNA profile. We also identified miR-29a-3p as an effective mediator for MAdCAM-1 expression. Administration of plasma exosome from *CrebH*^−/−^ mice led to prominent inflammatory signals in the liver of WT mice with inflammatory bowel disease (IBD).

**Conclusions:**

*CrebH* deficiency led to increased susceptibility to IBD-induced liver diseases via enhanced expression of adhesion molecules and concomitant infiltration of T lymphocytes. Exosomes can contribute to the progression of IBD-induced liver injury in *CrebH*^−/−^ mice. These study provide novel insights into the role of CrebH in IBD-induced liver injury.

**Supplementary Information:**

The online version contains supplementary material available at 10.1186/s13578-023-01065-9.

## Background

Inflammatory bowel diseases (IBDs), including Crohn’s disease (CD) and ulcerative colitis (UC), are associated with the destruction of gut structure and function, resulting in chronic intestinal inflammation [1]. Genetic and environmental factors are implicated in the immunopathologic process of IBD, leading to chronic inflammation in the gut [2]. Pathogenesis of IBD is closely related to an aberrant local immune response to intestinal microflora and uncontrolled endogenous regulator mechanisms [3, 4]. In addition, IBDs are considered systemic diseases because their symptoms occur in the gastrointestinal tract and cause problems outside the gut, commonly called extraintestinal manifestations (EIMs) [5]. However, the pathogenic factors leading to EIMs are not fully understood.

The liver is a critical site for antigen exposure and the response to invading pathogens during IBD. Liver-associated EIMs include primary sclerosing cholangitis (PSC), hepatitis, hepatic cirrhosis, fatty liver, and cholelithiasis, resulting in a high incidence of biliary cancer and colorectal cancer [6]. Among them, PSC is a severe IBD manifestation characterized by increased inflammation of the intrahepatic and extrahepatic bile ducts [7–9]. The prevalence of PSC patients suffering from concurrent IBD is approximately 70%, whereas the rate of PSC occurrence among IBD patients is only 1%–5% [10]. Although the mechanistic links between IBD and PSC remain largely unknown, a widely accepted hypothesis is translocation of microbiota and their products from the gut to the liver, triggering an aberrant cholangiocytic response [11]. Another hypothesis is that the proportion of T-lymphocytes expressing α4β7 integrin increases in the liver of humans and animals with PSC [12, 13]. In the gut, lymphocyte infiltration and activation are essential to protect the intestine from invading pathogens and play key roles in IBD pathogenesis [14]. Association of adhesion molecules with their ligand leads to tissue-specific trafficking of lymphocytes. Mucosal vascular addressin cell adhesion molecule 1 (MAdCAM-1), a gut-specific adhesion molecule, is a key player in lymphocyte trafficking with the vascular cell-adhesion molecule 1 (VCAM1) and intercellular adhesion molecule 1 (ICAM1) [15, 16]. In the liver, MAdCAM-1 is commonly detected in the hepatic sinusoids and has an important role in recruiting lymphocytes [13]. Although vascular adhesion protein 1 (VAP1) may regulate MAdCAM-1 expression in the liver [17], the regulation of MAdCAM-1 expression in the liver of PSC patients remains unclear.

Cyclic adenosine monophosphate (cAMP)-responsive element-binding protein H (CrebH, known as CREB3L3) is a transcription factor related to a member of the CREB/ATF family [18]. CrebH was initially known as a liver-specific transcription factor owing to its marked expression in the liver; its roles in hepatic glucose and lipid metabolism are commonly studied [19]. The expression and role of CrebH in the small intestine have been reported recently [20]. Therefore, we hypothesized that CrebH plays an important role in the gut–liver axis.

## Methods

### Animal studies

All mice were maintained at a constant temperature (20–22 ℃) under scheduled light: dark conditions (12:12 h); this animal study was approved by the guidelines of the Institutional Animal Care and Use Committee of the Korea Research Institute of Bioscience and Biotechnology (KRIBB-AEC-21129). We selected 10-week-old C57BL/6 J and *CrebH*^−/−^ male mice to be administered with 2.5% (w/v) dextran sulfate sodium (DSS, molecular weight = 36–50 kDa; MP Biomedicals, CA, USA) supplemented in drinking water to generate UC-mimetic animal models. For the CD model, anesthetized male mice were intrarectally injected with 3 mg dinitrobenzene (DNBS, St. Louis, MO, USA) in 100 μL 50% ethanol (EtOH). For the PSC animal model, 8-week-old mice were fed a 3.5-dieythioxycarbonyl-1,4-dihydrocollidine (DDC, Sigma-Aldrich, St. Louis, MO, USA) diet (standard rodent diet supplemented with 0.1% [w/w] DDC) for 7 days. WT male mice were twice injected with exosome (200 μg protein concentration/mice) isolated from WT and *CrebH*^−/−^ male mice treated with DSS for 7 days and then administered with 2.5% DSS for 7 days.

### Cell culture and establishment of stable cells

HepG2 and bEnd.3 cell lines purchased from ATCC were cultured in Dulbecco’s modified Eagle’s medium (DMEM; HyClone, Logan, UT, USA) containing 10% fetal bovine serum (FBS, HyClone), 100 U/mL penicillin, and 100 μg/mL streptomycin (Gibco, MA, USA) at 37 ℃ and 5% CO_2_. HepG2 cell lines constantly expressing the *CrebH* gene were generated by transfection with pcDNA3-Flag-*CrebH* [21] using the Lipofectamine LTX plus reagent system (Invitrogen, CA, USA) and selected by adding G418 solution (Sigma-Aldrich).

### Cell transfection and experiments

bEnd.3 cells were seeded in a 6 cm dish and cultured at 37 ℃ in a 5% CO_2_ incubator overnight. The cells were replaced with fresh complete media and transfected with miR-29a-3p mimic (Bioneer, Korea) and negative control (Bioneer) at 250 pM (final concentration) using RNAiMAX reagents (Thermo Fisher Scientific, MA, USA) according to the manufacturer’s protocol. After additional incubation for 24 h, the cells were treated with 50 ng/mL TNFα or vehicle and harvested after further 24 h incubation. The miR-29a-3p mimic sequence was 5′-ACUGAUUUCUUUUGGUGUUCAG-3′.

### Plasma analysis

Plasma alanine aminotransferase (ALT) and aspartate aminotransferase (AST) concentrations were measured automatically using a chemical analyzer (Hitachi, Tokyo, Japan). Alkaline phosphatase (ALP) levels were measured using an ALP assay kit (Abcam, MA, USA) according to the manufacturer’s protocol [22].

### Histological analysis

The colon, ileum, and liver tissues were cut at 5 μm thickness and stained separately with hematoxylin and eosin (H&E), similar to a previous study [23, 24]. The severity of colitis was blindly assessed by a pathologist and scored using the histological scoring method [23]. For immunohistochemistry, the slides were incubated with a boiled citrate solution for 15 min, blocked for 1 h, and further incubated with primary antibodies against MAdCAM-1 (MyBioSource, CA, USA), cleaved caspase 3 (Cell Signaling, MA, USA), and CD3 (Abcam) at 4 ℃ overnight. Then, slides were washed with PBS and stained with secondary antibodies and a Vectastain Elite ABC kit (Vector Labs, Burlington, ON, Canada) according to the manufacturer’s protocol. The positive cells were detected using diaminobenzidine (DAB, Vector Labs) and mounted with the antifade agent (Sigma-Aldrich). Tunel staining was performed according to the manufacturer’s manual.

### Myeloperoxidase (MPO) activity

MPO activity in the livers of WT and *CrebH*^−/−^ mice was measured using an MPO activity colorimetric assay kit (Biovision Inc., CA, USA), according to the manufacturer’s protocol [25].

### RNA isolation and quantitative real time-PCR

Total RNA was prepared using TRIzol reagent (Thermo Fisher) according to the manufacturer’s protocol. Complementary DNA (cDNA) was synthesized using 1 μg template RNA and an iScript^™^ cDNA Synthesis Kit (Bio-Rad, Hercules, CA, USA). Relative gene expression was determined using AccuPower 2 × Greenstar qPCR Master Mix (Bioneer) and a StepOnePlus^™^ Real-Time PCR device (Applied Biosystems, CA, USA). Each gene was normalized to 18 s rRNA. The primers used are listed in Additional file 1: Table S1. A thermal cycler was set for 40 cycles at an annealing temperature of 60 ℃.

### Fluorescence-activated cell sorting (FACS) analysis

Liver cells were prepared by passing through a 70 μm Falcon^™^ Cell strainer (Life sciences, MA, USA) and centrifugation of the supernatant into 40% Percoll (GE Healthcare, UK). The isolated cells were labeled with fluorophore-conjugated antibodies against PE-CD45 (BioLegend, CA, USA), FITC-CD3ε (BD Pharmingen, CA, USA), APC-CD8a (BD Pharmingen), and PerCP/Cy5.5-CD4 (BioLegend) for 30 min at 4 ℃. Labeled cells were assayed using a Gallio^™^ Flow Cytometer (Beckman Coulter, FL, USA). Data were analyzed using the FlowJo software (TreeStar, CA, USA).

### Western blotting

The samples were homogenized in RIPA buffer (Sigma-Aldrich) supplemented with a protease inhibitor (Roche Applied Science, Germany). The proteins were separated by electrophoresis on a 10%–12% sodium dodecyl sulfate–polyacrylamide gel, transferred to PVDF membranes, and blocked in TBST buffer with 5% skim milk. The membranes were incubated with primary antibodies against MAdCAM-1 (BD Biosciences, San Diego, CA, USA), α-tubulin (Cell Signaling), AKT1 (Cell Signaling), AKT2 (Cell Signaling), and TNF-R1 (Cell Signaling).

### Exosome isolation

Plasma exosome isolation was performed using the ExoQuick exosome precipitation kit (SBI System Biosciences, Mountain View, CA, USA) according to the manufacturer’s protocol [26, 27]. HepG2 cell lines constantly expressing CrebH were seeded in a 10 cm culture dish overnight and changed with fresh DMEM containing 10% EV-depleted FBS (SBI System Biosciences) for 48 h. The culture medium was centrifuged at 1000 × *g* for 5 min at 4 ℃ to eliminate suspended cells, filtered through a 0.22 μm syringe filter, and transferred to a Macrosep 100 KD filter system (PALL Laboratory, MA, USA) to enrich the particles with 30–90 nm molecular size. The exosomes contained in the enriched particles were isolated using ExoQuick-TC exosome precipitation solution (SBI System Biosciences). The exosome pellet was resuspended in 100 μL filtered-PBS and stored at − 80 ℃.

### Exosome characterization and treatment

The exosome size was determined using a dynamic light scattering system (Otsuka ELS-Z, Japan). Exosomes isolated from the plasma of WT and *CrebH*^−/−^ mice were quantified using an ExoELISA-ULTRA assay kit (SBI System Biosciences) according to the manufacturer’s guidelines [28]. Exosomal markers were estimated by immunoblotting using antibodies against TSG101 (Abcam), CD9 (Abcam), and Histone H3 (Sigma-Aldrich). Exosomes were labeled using an ExoGlow-Membrane EV labeling kit (SBI System Biosciences) following the manufacturer’s instructions. Labeled exosomes were cocultured with bEnd.3 cell lines for the indicated time. Images were obtained using fluorescence microscopy (Olympus, Tokyo, Japan). Exosomal proteins were quantified using Bradford protein assay and bEnd.3 cells were treated with exosomes (30 µg/mL protein concentration contained in exosome) for 24 h.

### Exosomal miRNA profiling

Exosomal total RNA was profiled using a NanoString nCounter Mouse miRNA Panel (NanoString Technologies, WA, USA) according to the manufacturer’s instructions [29]. Each RNA sample (50 ng) was added to the miRNA-tag ligation reaction. Ligated miRNA was diluted (1:5), added to hybridization, and subjected to 3 h of automated processing per cartridge. The acquired data were normalized by a set of six positive and negative control probes included in the system and processed using nSolver software (version 4.0, NanoString Technologies). Relative miRNA was expressed as fold-change.

### Transcriptome analysis

As described above, total RNA was isolated from liver tissues of WT-DSS and *CrebH*^−/−^-DSS or control and DDC-treated mice. Messenger RNA was purified from total RNA using poly-T oligo-attached magnetic beads. RNA-sequencing (RNA-seq) libraries were constructed and sequenced on an Illumina X Ten. The number of reads mapped was counted using featureCounts (v1.5.0-p3); then, the fragments per kilobase of transcript per million mapped reads of each gene was calculated based on the length of the gene. Differential expression of each group was performed using the DESeq2 R package (v1.20.0). The resulting P values were adjusted using Benjamini and Hochberg’s approach [30], and genes with an adjusted P-value < 0.05 were assigned as differentially expressed. Gene ontology (GO) analysis using the WEB-based Gene SeT AnaLysis Toolkit (https://www.webgestalt.org) was applied to analyze the molecular functions of overlapped genes with significant differences. Heatmaps were generated by the TreeView3 program (https://bitbucket.org/TreeView3Dev/treeview3).

### Statistical analysis

Data were analyzed using the GraphPad Prism software (version 8.0; San Diego, CA, USA) and expressed as mean ± standard error of the mean (SEM). Differences between the two groups were analyzed using the Student’s *t*-test. Survival differences between groups were analyzed using the log-rank (Mantel–Cox) test. Statistical significance was set at *P*-value < 0.05.

## Results

### CrebH deficiency does not affect the development of IBD pathogenesis

**Fig. 1:**
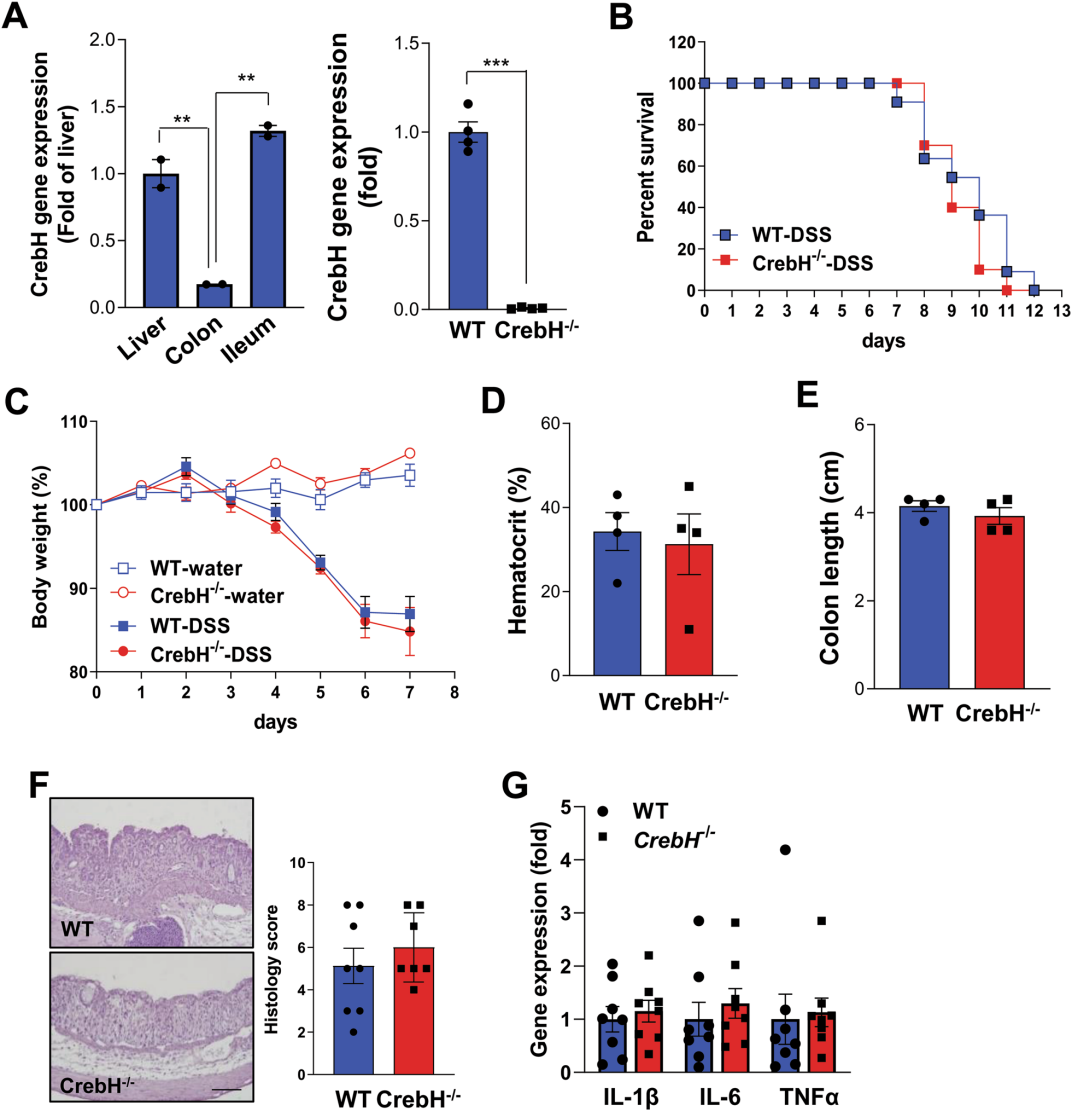
Expression of CrebH mRNA and effects of CrebH ablation on the progression of IBD pathogenesis. **A** Expression of CrebH mRNA was determined in the colon and ileum by RT-qPCR (left). The specificity of the primer set for CrebH was validated by RT-qPCR in the liver from WT and *CrebH*^*−/−*^ mice (right). **P < 0.01 or ***P < 0.001. **B** Survival differences of WT and *CrebH*^*−/−*^ mice after 2.5% DSS were monitored daily. WT (n = 11) and *CrebH*^*−/−*^ (n = 10). **C** Bodyweight loss was estimated in the mouse groups; WT-con (n = 4, open square), *CrebH*^*−/−*^-con (n = 4, blue square), WT-DSS, (n = 4, open circle), and *CrebH*^*−/−*^-DSS (n = 4, red circle). **D**, **E** Hematocrit and colon length of WT (n = 4) and *CrebH*^*−/−*^ (n = 4) mice. **F** Liver histopathology of WT (n = 8) and *CrebH*^*−/−*^ (n = 7) mice were estimated by hematoxylin and eosin (H&E) staining (left) and scoring (right). The scale bar represents 200 μm. **G** The levels of *IL-1β*, *IL-6*, and *TNFα* were measured by qRT-PCR in the colon tissues from WT (blue) and *CrebH*^*−/−*^ (red) mice treated with 2.5% DSS for 7 days. Statistical analysis was performed using two-tailed Student’s *t*-test. Error bars represent the mean ± SEM

CrebH is highly detected in both the liver and small intestine [19, 20]. To confirm this observation, mRNA expression of *CrebH* was evaluated by RT-qPCR in ileum and colon tissues. High levels of CrebH were observed in the ileum, while its expression was very rare in the colon (Fig. 1A). The specificity of the PCR for CrebH was validated by using liver samples from WT and *CrebH*^−/−^ mice (Fig. 1A). Firstly, the ablation effects of CrebH in DSS-induced colitis were determined. DSS administration to WT and *CrebH*^−/−^ mice led to death and the development of severe pathogenesis in colon tissues, as evidenced by body weight loss, reduced hematocrit percentage and colon length, and severe inflammation with no significant differences between WT and *CrebH*^−/−^ mice (Fig. 1B–F). Consistently, pro-inflammatory cytokine expression did not differ between the groups (Fig. 1G), suggesting that rare expression of *CrebH* in the colon is not enough to alter the pathogenesis of UC. Similar to the UC model, in CD animal models, CrebH did not affect IBD progression, as evidenced by the lack of significant differences (Additional file 2: Fig. S1A–C).

### Ablation of CrebH deteriorates a liver injury of mice with IBD

**Fig. 2:**
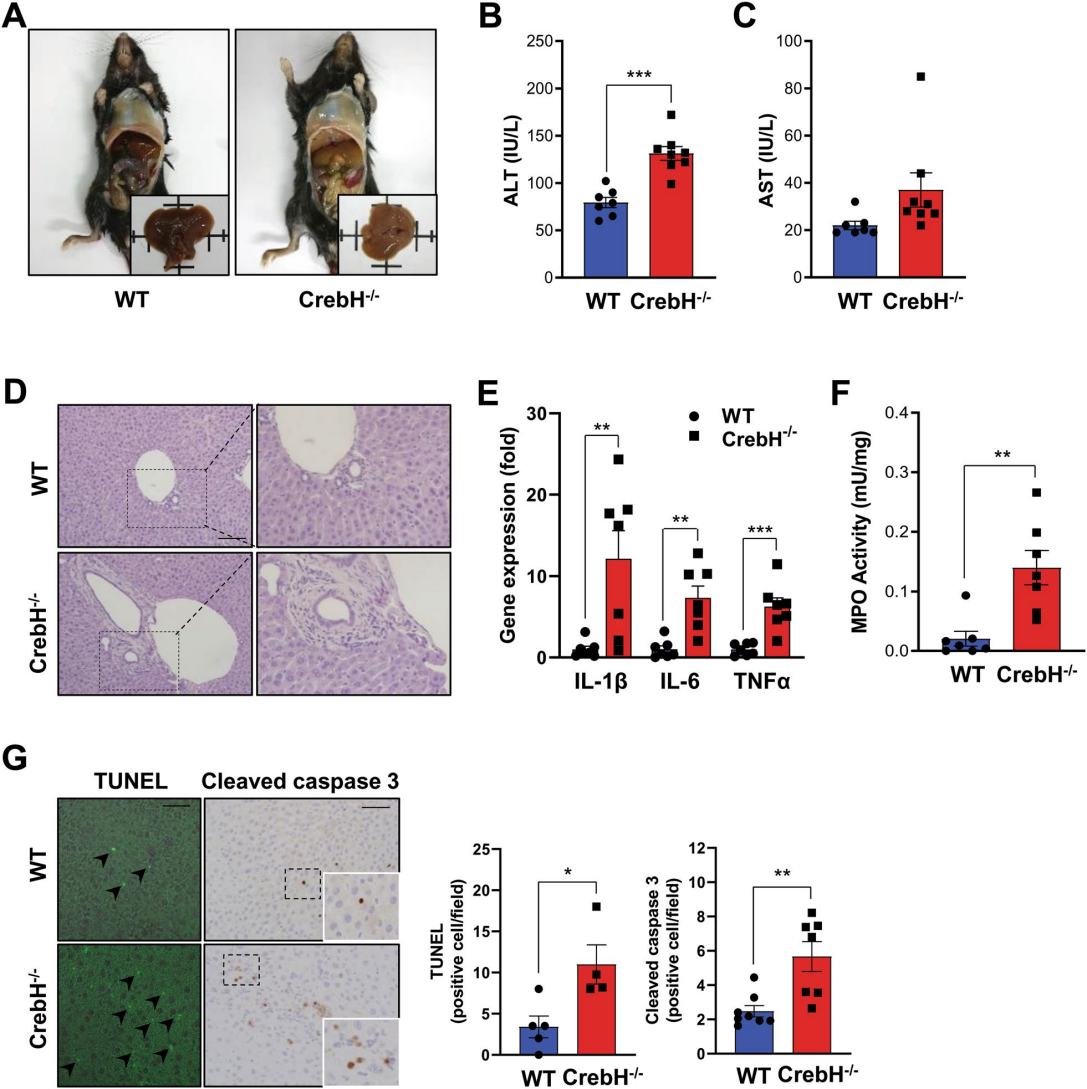
CrebH deficiency accelerates IBD-associated liver damage. **A** Representative liver aspect of WT and *CrebH*^*−/−*^ mice treated with 2.5% DSS for 7 days. **B**, **C** ALT and AST levels were measured in the plasma of WT (n = 7) and *CrebH*^*−/−*^ (n = 8) mice. ***P < 0.001. **D** Representative liver histology of WT and CrebH^−/−^ mice were stained with H&E. The bar represents 200 μm. **E** The levels of *IL-1β*, *IL-6*, and *TNFα* in the liver from WT (n = 7) and *CrebH*^*−/−*^ (n = 7) mice were analyzed by qRT-PCR. **P < 0.01 or ***P < 0.001. **F** Liver MPO activity was measured using a commercially available assay kit. **P < 0.01. **G** Liver images of tunel (left images) and cleaved caspase 3 (right graph) in WT and *CrebH*^*−/−*^ mice. The scale bar represents 200 μm. *P < 0.05, **P < 0.01. Positive cells per field were counted using the ImageJ software. Statistical analysis was performed using a two-tailed Student’s *t*-test. Error bars represent the mean ± SEM

IBD leads to concomitant disease development outside the gut [31]. Therefore, we estimated the liver pathology to confirm the role of CrebH in IBD-associated liver damage. The liver of *CrebH*^−/−^ mice after administering DSS showed a pale color compared with those of WT mice (Fig. 2A). Levels of ALT and AST, two liver damage markers, were increased in the plasma of *CrebH*^−/−^ mice compared with that in the plasma of WT mice (Fig. 2B, C). Excitingly, liver histology of *CrebH*^−/−^ mice displayed enlarged bile ducts (Fig. 2D). In contrast to the gut data, the expression of pro-inflammatory cytokines, such as *IL-1β*, *IL-6*, and *TNFα,* was significantly increased in the livers of *CrebH*^−/−^ mice compared with WT mice (Fig. 2E). Furthermore, MPO activity was more enhanced in the livers of *CrebH*^−/−^ mice than in those of WT mice (Fig. 2F). These data show that *CrebH*^−/−^ mice have high inflammatory conditions in their livers. Furthermore, apoptotic cells significantly increased in the livers of *CrebH*^−/−^ mice (Fig. 2G). Consistent with DSS-liver, in the CD mouse model, *CrebH*^−/−^ mice showed severe pathogenesis compared with WT mice (Additional file 3: Fig. S2A–D).

### Loss of CrebH leads to enhanced CD8^+^***T lymphocyte infiltration ***via*** up-regulation of adhesion molecules in the liver***

**Fig. 3:**
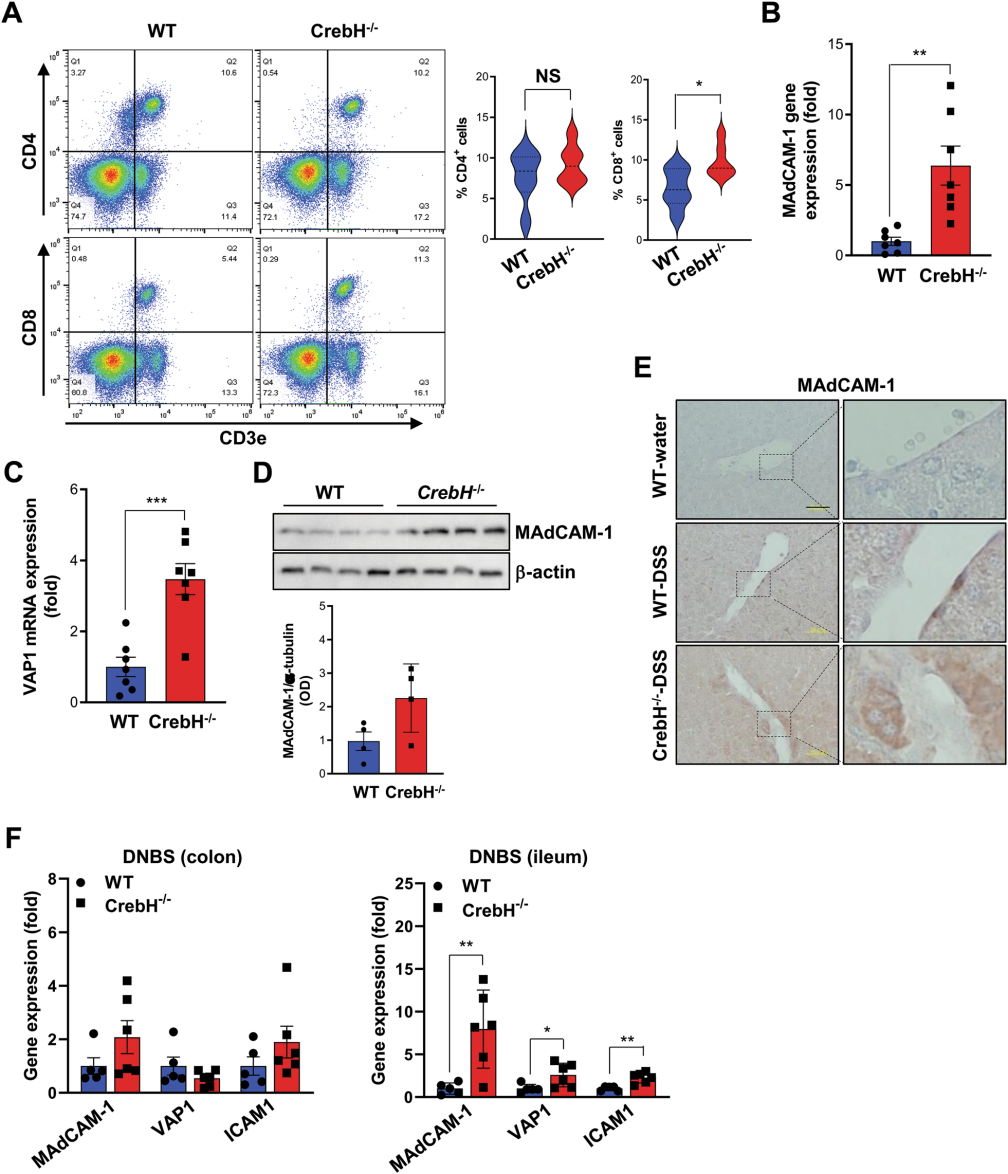
CrebH deficiency leads to increased T lymphocyte infiltration via enhanced adhesion molecules in the liver. **A** Cd3^+^Cd4^+^ and Cd3^+^Cd8^+^ double-positive cells were determined by flow cytometric analysis of cell population in the liver of WT (n = 6) and *CrebH*^*−/−*^ (n = 6) mice after DSS treatment. *P < 0.05. **B**, **C** mRNA levels of *MAdCAM-1* and *VAP1* in the liver from WT and *CrebH*^*−/−*^ mice were measured by qRT-PCR. **P < 0.01, or ***P < 0.001. **D** Protein levels of MAdCAM-1 in the liver from WT and *CrebH*^*−/−*^ mice were evaluated by immunoblotting using specific antibodies against each protein, and their optical intensities were normalized by β-actin. *P < 0.05. **E** Representative immunohistochemistry images on MAdCAM-1 in the liver from WT and *CrebH*^*−/−*^ mice. The scale bar represents 200 μm. **F** Gene expression of *MAdCAM-1*, *VAP1*, and *ICAM1* was estimated in the colon (left) and ileum (right) of WT (n = 5) and *CrebH*^*−/−*^ (n = 6) mice treated with DNBS for 2 days. *P < 0.05, **P < 0.01. Statistical analysis was performed using a two-tailed Student’s *t*-test. Error bars represent the mean ± SEM

In IBD-associated liver disease, T lymphocytes infiltrate the liver and play a critical role in hepatic duct inflammation [16]. FACS analysis showed that CD8^+^ T lymphocytes were significantly upregulated in the liver of *CrebH*^−/−^ mice after DSS treatment, whereas CD4^+^ T lymphocyte regulation was not significantly altered (Fig. 3A). MAdCAM-1 and *VAP1*, aberrantly expressed in the liver of PSC patients and animal models with IBD, were significantly increased in the livers of *CrebH*^−/−^ mice compared to WT mice after DSS administration (Fig. 3B–D). Histological data show that MAdCAM-1 was primarily distributed in the endothelium of vessels or sinusoids after DSS treatment (Fig. 3E). CrebH is mostly expressed in the small intestine, and we investigated the MAdCAM-1 mRNA expression in ileum and colon tissues of mice with CD to determine the CrebH dependency on MAdCAM-1 expression. Consistent with *CrebH* expression levels, differential expressions of adhesion molecules containing MAdCAM-1 were more prominent in the small intestine than the large intestine (Fig. 3F), explaining the CrebH contribution to local expression of *MAdCAM-1* and the lack of difference in pathogenesis in the large intestines of both groups.

### Different gene expressions in the liver of CrebH^***−/−***^*** mice are closely related with PSC-liver***

**Fig. 4:**
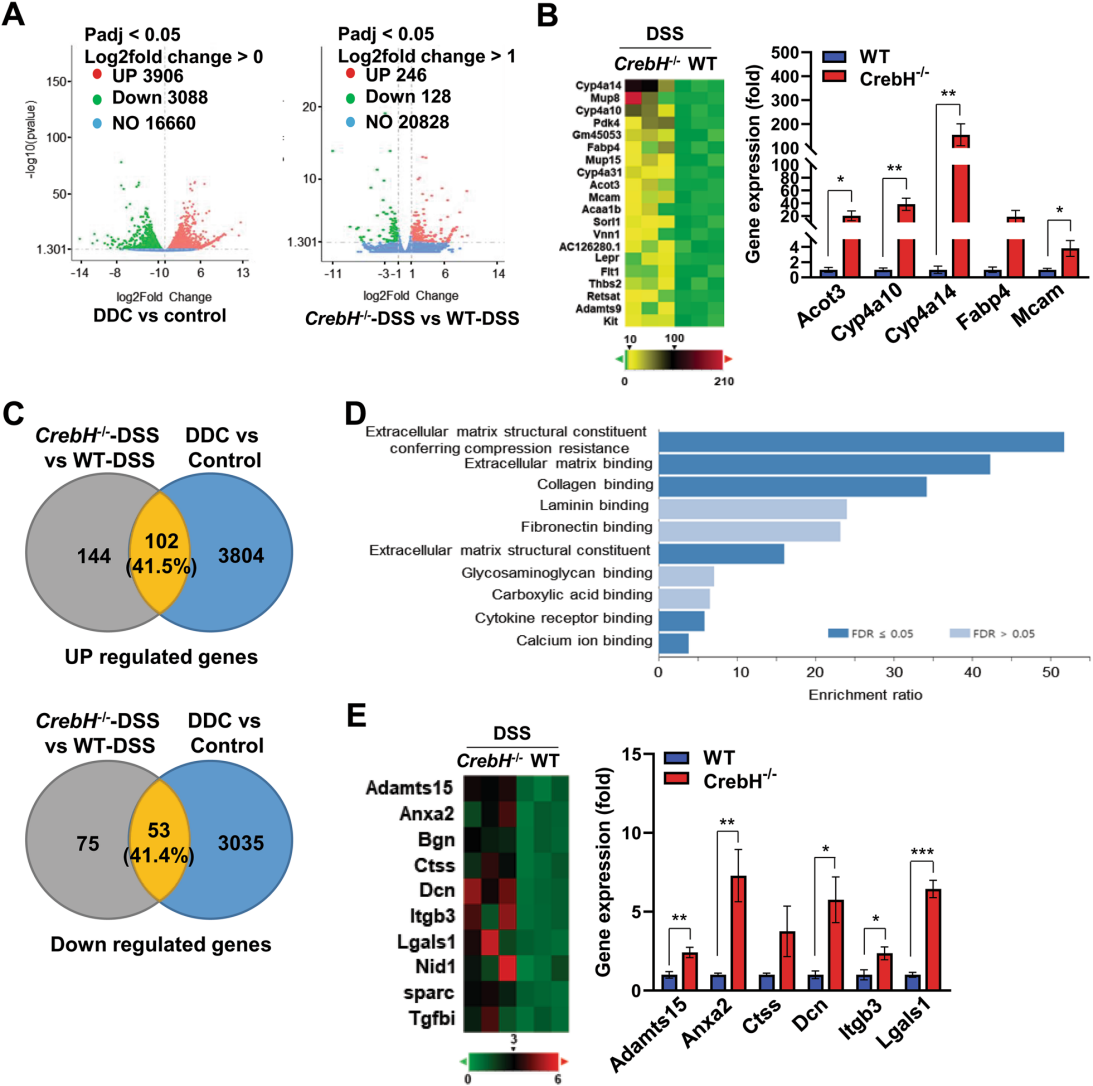
Gene expressions in the liver of *CrebH*^*−/−*^ mice overlap with those expressed differentially in mice with PSC. **A** Volcano plot of genes significantly altered between DDC (n = 3) vs. control groups (n = 3) and *CrebH*^*−/−*^-DSS (n = 3) vs. WT-DSS (n = 3). **B** Heatmap (left, RNA-seq data) showing top 20 genes significantly upregulated in the liver of *CrebH*^*−/−*^-DSS mice compared with WT-DSS mice; several genes were validated by qRT-PCR. WT (n = 5) and *CrebH*^*−/−*^ (n = 5) mice. *P < 0.05 or **P < 0.01. Acot3, acyl-CoA thioesterase 3; Cyp4a10, cytochrome P450 4A10; Cyp4a14, cytochrome P450 omega-hydroxylase 4a14; Fabp4, fatty acid-binding protein 4; Mcam, melanoma cell adhesion molecule. **C** Comparison of transcriptome analysis of DEGs between *CrebH*^*−/−*^-DSS vs. WT-DSS and DDC vs. control groups. Venn diagram of overlapping differentially upregulated (upper) and downregulated (lower) gene expression. **D** Top 10 enriched in “molecular function” of the RNA-seq dataset. GO enrichment of DEGs was analyzed using the WEB-based Gene SeT AnaLysis Toolkit (www.webgestalt.org). **E** Heatmap of genes enriched in extracellular matrix binding category; several genes were validated by qRT-PCR. *P < 0.05, **P < 0.01, ***P < 0.001. Adamts15, ADAM metallopeptidase with thrombospondin type 1 motif 15; Anxa2, annexin A2; Ctss, cathepsin S; Dcn, decorin; Itgb3, integrin subunit beta 3; Lgals1, galectin 1. Statistical analysis was performed using a two-tailed Student’s *t*-test. Error bars represent the mean ± SEM

As described, we observed that the livers of *CrebH*^−/−^ mice after DSS have similar histological and pathogenic characteristics as PSC. Interestingly, *CrebH*^−/−^ mice were more susceptible to the pathogenesis of PSC caused by DDC treatments than were WT mice (Additional file 4: Fig. S3). Therefore, we performed differential gene expression analysis to estimate the similarities between *CrebH*^−/−^ mice liver injury caused by DSS and PSC-liver. Gene expression analyzed by RNA-seq in the liver of *CrebH*^−/−^-DSS vs. WT-DSS groups was compared with the dataset of DDC-treated vs. control groups. The volcano plot of differentially expressed genes (DEGs) shows significant genes, including 3906 upregulated and 3,088 downregulated genes in DDC vs. control groups and 246 upregulated and 128 downregulated genes in *CrebH*^−/−^-DSS vs. WT-DSS (Fig. 4A). The heatmap showed the top 20 genes upregulated in the livers of *CrebH*^−/−^-DSS compared to those of WT-DSS and several genes were validated by qRT-PCR (Fig. 4B). Excitingly, 41.5% upregulated and 41.4% downregulated genes in *CrebH*^−/−^-DSS vs. WT-DSS overlapped with genes altered in DDC vs. control, respectively (Fig. 4C). On analyzing the gene ontology terms, the three most enriched gene sets in molecular function (MF) terms of genes that overlapped between the genes increased in the *CrebH*^−/−^-DSS compared to the WT-DSS and in the DDC group compared to the control group were “extracellular matrix structural constituents conferring compression resistance,” “extracellular matrix binding,” and “collagen-binding,” which are closely related to tissue fibrosis (Fig. 4D). Among these gene set in Fig. 4D, the 10 genes associated with extracellular matrix binding, including *Adamts15*, *Anxa2*, *Bgn*, *Ctss*, *Dcn*, *Itgb3*, *Lgals1*, *Nid1*, *Sparc*, and *Tgfbi*, were displayed in the heatmap (Fig. 4E, *left*), and several genes validated by qRT-PCR showed a similar pattern with RNA-seq analysis (Fig. 4E, *right*). These data suggest that liver damage in *CrebH*^−/−^ mice induced by DSS administration included important pathological features of PSC liver.

### Exosomes play an important role in regulating adhesion molecules during liver injury of CrebH^***−/−***^*** mice***

**Fig. 5:**
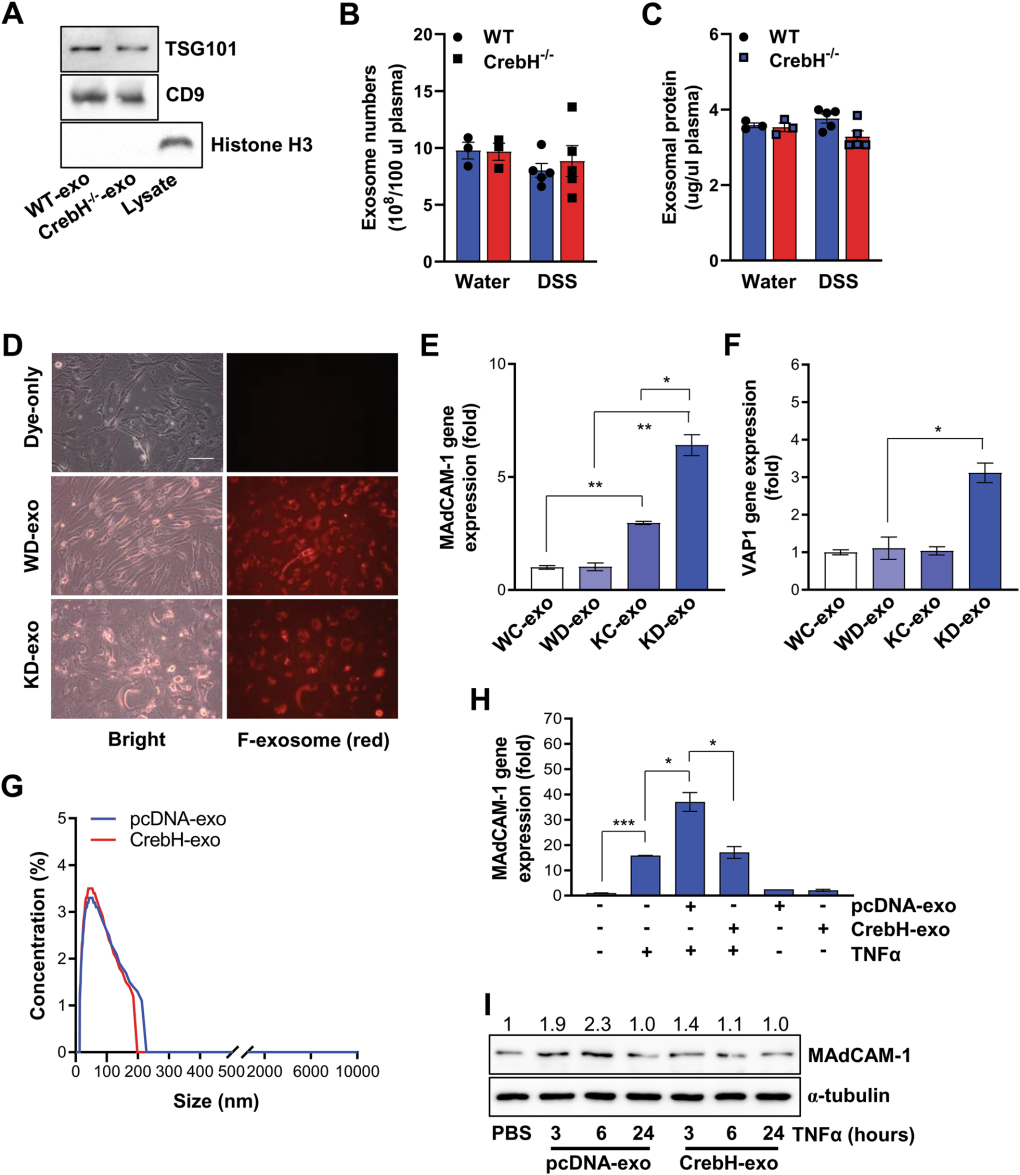
Exosomes isolated from plasma of both mouse groups or culture media of cells expressing exogenous CrebH stimulate the expression of *MAdCAM-1* and *VAP1* in endothelial cell lines. **A** Immunoblot data for TSG101 and CD9 of exosomes. Histone H3 was used as a negative control for exosomes and lysate was from colon tissue. **B** Exosome numbers isolated from WT (blue) and *CrebH*^*−/−*^ (red) mice were estimated using a commercially available exosome quantification kit in 100 μL plasma. **C** Exosomal protein levels were measured by the Bradford protein quantification method. Water (n = 3) and DSS (n = 5). **D** Exosome uptake was investigated in bEnd.3 cells. **E**, **F** Expressions of *MAdCAM-1* and *VAP1* response to exosome isolated from WT and *CrebH*^*−/−*^ mice. WT-control exosome (WC-exo), WT-DSS exosome (WD-exo), *CrebH*^*−/−*^-control exosome (KC-exo), or *CrebH*^*−/−*^-DSS exosome (KD-exo) groups. *P < 0.05 or **P < 0.01. **G** Size distribution of exosomes isolated from the culture medium was determined by a dynamic light scattering system. **H** TNFα-induced *MAdCAM-1* expression was estimated by qRT-PCR after treatment of pcDNA–exo or CrebH-exo. *P < 0.05 or ***P < 0.001. **I** Protein levels of MAdCAM-1. Alpha-tubulin was used as a loading control. Statistical analysis was performed using a two-tailed Student’s *t*-test. Error bars represent the mean ± SEM

To identify systemic mediators affected by CrebH ablation, we performed multiplex plasma analysis. No significant differences between *CrebH*^*−*/−^ and WT mice were detected in the plasma (Additional file 5: Table S2). Exosomes play an important role in cell-to-cell communication in systemic and local systems [32]. Therefore, we hypothesized that exosomes might be potential mediators contributing to the aberrant pathogenesis in *CrebH*^−/−^-DSS mice. First, we characterized exosomes isolated from the plasma of each mice group to estimate their successful isolation (Fig. 5A–D). bEnd.3 cells, an endothelial cell line, were stimulated with exosomes isolated from the plasma of WT-water exosome (WC-exo), WT-DSS exosome (WD-exo), *CrebH*^−/−^-water exosome (KC-exo), and *CrebH*^−/−^-DSS exosome (KD-exo). KC-exo treatment led to significantly higher *MAdCAM-1* expression than WC-exo treatment (Fig. 5E). Furthermore, KD-exo additionally elevated the effects of KC-exo (Fig. 5E). VAP1 expression was upregulated only in KD-exo-treated cells (Fig. 5F). To further investigate the liver-specific effects of CrebH, HepG2 cell lines constantly overexpressing *CrebH* were generated (Additional file 6: Fig. S4). Isolated exosomes from the medium of the stable cells were characterized (Fig. 5G). Consistent with another study [33], TNFα treatment stimulated *MAdCAM-1* expression, and co-treatment with pcDNA-exo significantly enhanced its expression in bEnd.3 cells (Fig. 5H). Interestingly, CrebH-exo eliminated pcDNA-exo-induced *MAdCAM-1* expression (Fig. 5H), suggesting that *CrebH* can modulate *MAdCAM-1* expression in an exosome dependent manner. Protein and mRNA levels for MAdCAM-1 further supported this result (Fig. 5I).

### Profiling of exosomal miRNA and identification of miR-29a-3p as an effector miRNA

**Fig. 6:**
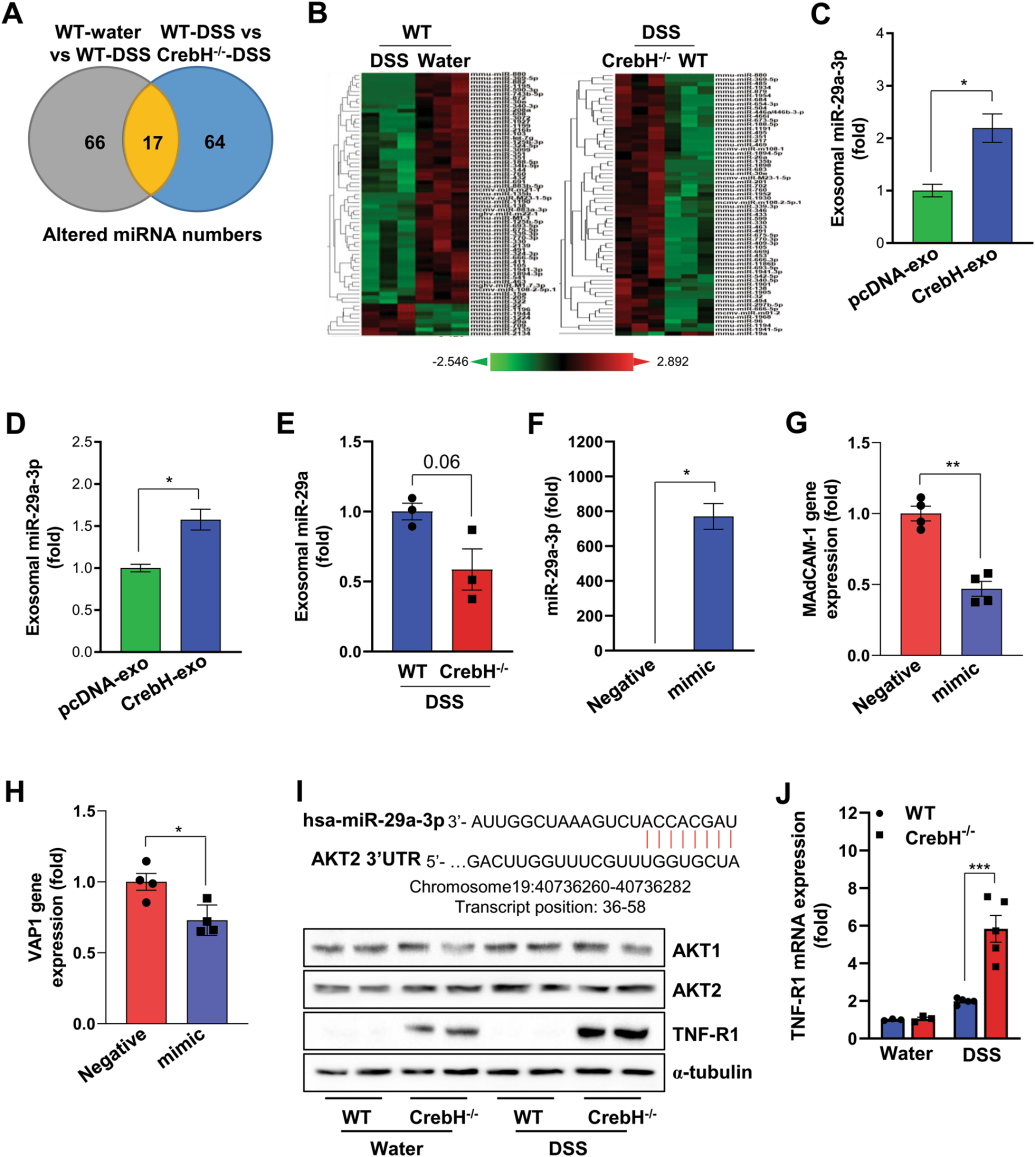
Exosomal miRNA from plasma of WT and *CrebH*^*−/−*^ mice and the effect of miR-29a-3p on expression of *MAdCAM-1* and *VAP1*. **A** Venn diagram of exosomal miRNA altered between WT-control vs. WT-DSS or WT-DSS vs. *CrebH*^*−/−*^-DSS. **B** Heatmaps showing hierarchical clustering of differentially expressed exosomal miRNA between DSS vs. control (left) and *CrebH*^*−/−*^-DSS vs. WT-DSS (right). **C** miR-29a-3p levels analyzed by miRNA profiling of stable HepG2 cells overexpressing pcDNA3 or CrebH. **D** Exosomal miR-29a-3p concentration was validated by qRT-PCR in exosomes released from HepG2 cells overexpressing pcDNA3.1 or CrebH. **E** miRNA profiling data for exosomal miR-29a levels of *CrebH*^*−/−*^-DSS compared with WT-DSS. **F** Effect of the mimic on miR-29a-3p expression in bEnd.3 cells. **G**, **H** Gene expression of *MAdCAM-1* and *VAP1* response to miR-29a mimic. **I** Target sequence on AKT 3′ UTR against miR-29a-3p (upper) and representative western blot data for AKT1, AKT2, and TNF-R1 (lower). J, TNF-R1 mRNA levels of each groups. *P < 0.05, **P < 0.01, or ***P < 0.001. Statistical analysis was performed using a two-tailed Student’s *t*-test. Representative data are from at least two independent experiments. Error bars represent the mean ± SEM

To identify exosomal mediators affecting liver pathogenesis during IBD, we performed miRNA profiling of exosomes using NanoString analysis. The Venn diagram revealed that altered miRNA between WC-exo and WD-exo shared 17 miRNAs compared with WD-exo and KD-exo (Fig. 6A). The heatmap shows aberrant expression of various miRNAs from each group (Fig. 6B and Additional file 7–9: Table S3–5). Excitingly, most altered miRNA was increased in the plasma of *CrebH*^−/−^ mice compared with that of WT after administration of DSS, suggesting that CrebH can regulate exosomal miRNA contents (Fig. 6B). To identify hepatocyte-specific mediators, we performed miRNA profiling in the exosomes derived from HepG2 cells constantly overexpressing pcDNA3 or CrebH and found miR-29a as a potential target miRNA. The sequence of mouse miR-29a was the same as that of humans and rats. miR-29a-3p was found in exosomes from HepG2 cells overexpressing CrebH protein with upregulated expression (Fig. 6C), and its levels were validated by qRT-PCR (Fig. 6D). In contrast, miR-29a-3p was downregulated in the plasma of *CrebH*^−/−^ mice compared with that in WT mice (Fig. 6E). Previously, Deng et al. reported that miR-29a-3p regulates several TNFα-induced adhesion molecules, including *VCAM1*, *ICAM1*, and *E-selectin*, by targeting TNF receptor-1 (TNF-R1) in various endothelial cell lines [34]. Therefore, we generated a miR-29a-3p mimic to investigate its effect on *MAdCAM-1* expression. Transfection of the mimic into bEnd.3 cells led to an approximately 700-fold increase in the miR-29a-3p level compared with the negative control (Fig. 6F). We found that the mimic inhibited *MAdCAM-1* and *VAP1* expression in bEnd.3 cells (Fig. 6G, H). Recent studies have suggested that *MAdCAM-1* expression is regulated by TNF-α and/or AKT signaling pathways [33]. We found a target sequence in the AKT2 3′-untranslated region (UTR; DIANA tools; http://diana.imis.athena-innovation.gr; Fig. 6I). Our western blot data showed that AKT2 levels were slightly higher in the liver of *CrebH*^−/−^ mice than in that of WT mice under healthy conditions (Fig. 6I). However, this pattern was not observed after DSS treatment. Interestingly, TNF-R1 levels were dramatically increased in the livers of *CrebH*^−/−^ mice, and this induction was more strongly enhanced by DSS administration (Fig. 6I). Furthermore, mRNA expression of TNF-R1 were upregulated in the liver of *CrebH*^−/−^ mice compared to WT mice after DSS administration (Fig. 6J). The data suggest that aberrant expression of adhesion molecules in the liver of *CrebH*^−/−^ mice might have occurred owing to increased TNF-R1.

### Plasma exosomes isolated from CrebH^***−/−***^*** mice with IBD aggravate DSS-induced liver injury in WT mice***

**Fig. 7:**
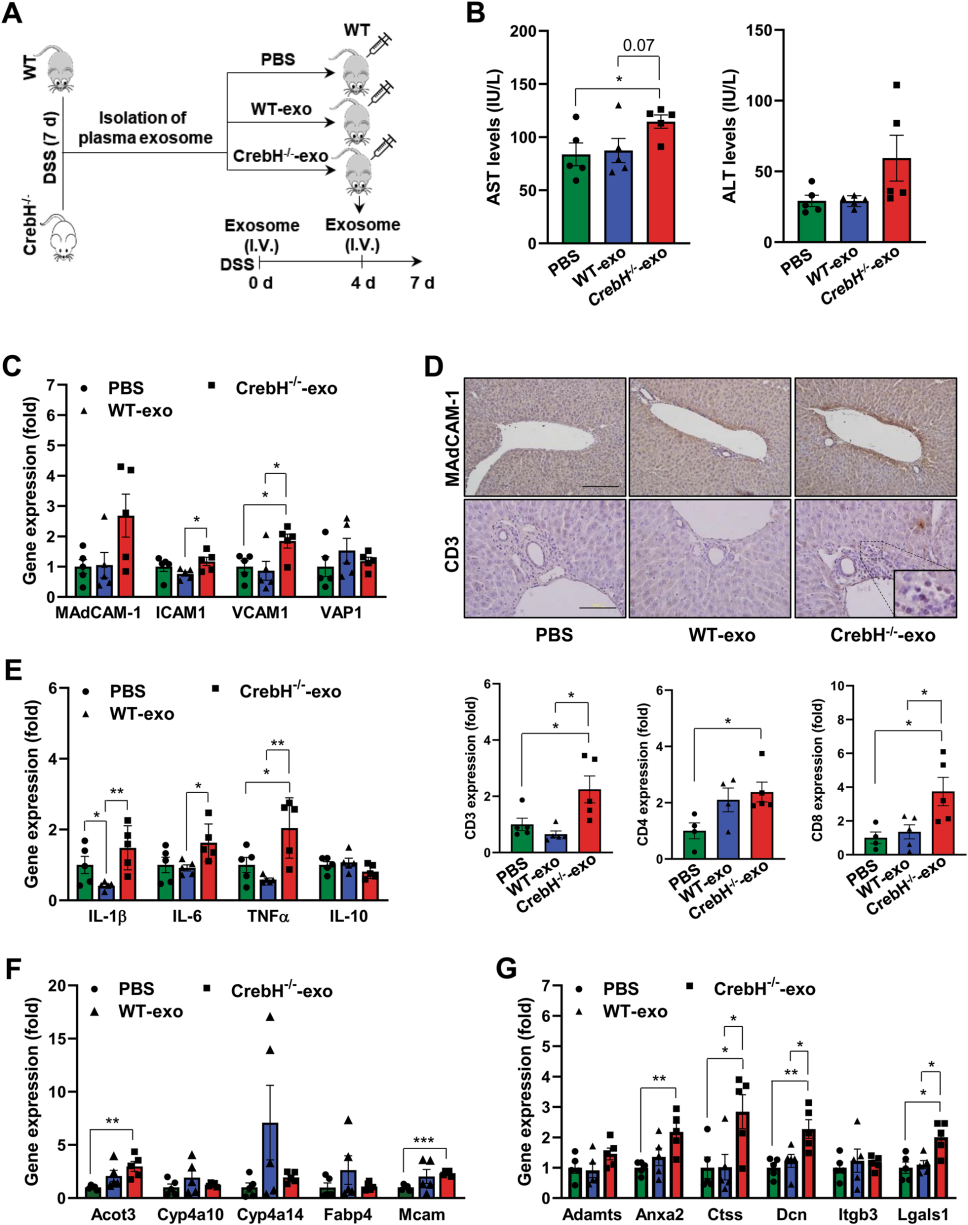
Plasma exosomes isolated from *CrebH*^*−/−*^* mice* with IBD stimulate hepatic inflammation in the liver of WT mice treated with DSS. **A** Schematic representation of the experimental schedule. Plasma exosomes from WT and *CrebH*^*−/−*^ mice after administration of DSS for 7 days were isolated and then WT mice were treated with isolated exosomes (200 ug protein concentration) at 0 and 4 days of DSS schedule. PBS were used as a control for exosome treatment. **B** AST and ALT levels. **C** Gene expression of *MAdCAM-1, ICAM1, VCAM1,* and *VAP* in the livers of each group. **D** Immunohistochemistry for MAdCAM-1 and CD3 (upper), confirmed by mRNA expression of CD3, CD4, or CD8 (lower). The scale bars represent 200 μm (MAdCAM-1) and 100 μm (CD3), respectively. **E** Gene expression of *IL-1β, IL-6, TNFα,* and *IL-10* in the livers of each group. **F** Gene expression of *Acot3, Cyp4a10, Cyp4a14, Fabp4,* and *Mcam* in the livers of each group. **G** Gene expression of *Adamts, Anxa2, Ctss, Dcn, Itgb3,* and *Lgals1* in the livers of each group. *P < 0.05, **P < 0.01, ***P < 0.001. Statistical analysis was performed using a two-tailed Student’s *t*-test. Error bars represent the mean ± SEM

To investigate the exosome effects against liver damages, plasma exosomes from WT and *CrebH*^−/−^ mice administered with DSS for 7 days were isolated and then injected into WT mice followed by DSS (Fig. 7A). Plasma AST levels were significantly increased in mice injected with *CrebH*^−/−^-exo compared with PBS controls (Fig. 7B). Although ALT levels were not significant, they increased in mice treated with *CrebH*^−/−^-exo compared with both controls (Fig. 7B). This suggests that the exosomes can contribute to the development of liver injury in WT mice. Adhesion molecules increased in the liver of *CrebH*^−/−^ mice were also up-regulated by *CrebH*^−/−^-exo without *VAP1* (Fig. 7C) and MAdCAM-1 staining intensity in the liver of mice treated with *CrebH*^−/−^-exo was stronger than in the DSS-PBS and WT-exo groups (Fig. 7D), resulting in enhanced hepatic infiltration of T lymphocytes evidenced by increased *CD3* positive cells and CD8 expression (Fig. 7D). Levels of proinflammatory cytokines were significantly higher in liver treated with *CrebH*^−/−^-exo compared with PBS and WT-exo controls (Fig. 7E). Interestingly, the expressions of *IL-1β* and *TNFα* showed a downward tendency in liver treated with WT-exo compared with PBS controls, providing a protective role of exosome against hepatic inflammation during IBD (Fig. 7E). Finally, we confirmed the DEGs observed in NGS dataset. The Acot3 and Mcam genes increased in *CrebH*^−/−^ mice compared with WT mice, and were significantly higher in mice treated with *CrebH*^−/−^-exo than in the only-PBS group; no significantly different expression was observed between mice with *CrebH*^−/−^-exo and those with WT-exo (Fig. 7F). Excitingly, Anxa2, Ctss, Dcn, and Lgals1 among DEGs overlapped with PSC-liver were significantly increased by treatment of *CrebH*^−/−^-exo compared with both control groups (Fig. 7G). As these genes are involved in extracellular matrix binding, our results might suggest that exosomes from *CrebH*^−/−^ mice are strongly associated with IBD induced-fibrosis.

## Discussion

Liver homeostasis is closely related to the gut environment because approximately 70% of blood derived from the gut reaches the liver [35]. This gut–liver axis has been implicated in various liver diseases such as PSC [36]. Membrane-bound transcriptional factor CrebH is primarily expressed in the liver and regulates genes related to triglyceride metabolism and fatty acid oxidation [19]. Recently, CrebH expression has also been reported in the small intestine [20]. However, the potential roles of CrebH in the gut–liver axis remain completely unknown. This study employed a genetic mouse model to determine the effects of *CrebH* ablation on IBD in the gut and liver. To the best of our knowledge, this study is the first to show the role of CrebH in IBD-induced liver injury.

PSC, a well-known clinically IBD-connected chronic liver disease, is evidence of an interaction between the gut and the liver [6]. Clinically, most PSC patients have concurrent IBD, while IBD patients have only 1%–5% PSC, suggesting that liver might have protective systems against IBD-induced PSC progression. PSC-liver is characterized by inflammation and onion skin-type fibrotic lesions around bile ducts, resulting in bile duct strictures and accumulation of bile acids into the liver [37]. Lymphocyte infiltration is a common characterization important for disease progression in IBD and IBD-related liver disorders [15, 16]. Interestingly, *CrebH*^−/−^ mice exhibited enlarged bile ducts, enhanced inflammation, and DEGs highly overlapped with PCS-liver. Furthermore, MAdCAM-1, demonstrated as a gut-specific adhesion molecule [12] and known as a marker for PSC liver [13], significantly increased in the liver of *CrebH*^−/−^ mice. Therefore, our study suggests that CrebH might be involved in the progression of IBD-related liver diseases such as PSC.

Reciprocal interactions between the gut and liver during IBD is established through the portal vein, which carries gut-origin products to the liver. The systemic effectors involved in liver injury progression during IBD contain various cytokines [38] and chemokines [39] together with bacteria and their products [40]. However, we did not find any differences in the plasma in our experimental conditions, suggesting another potential factors regulating liver injury. Exosomes play a critical role in cell-to-cell communication and protect their contents, including protein, RNA, DNA, and metabolites in the blood system [32]. Therefore, we hypothesized that exosomes might play an important role in the accelerated pathogenesis of the liver in *CrebH*^−/−^ mice. Fortunately, exosomes isolated from the plasma of *CrebH*^−/−^ mice can effectively stimulate *MAdCAM-1* and *VAP1* expression in bEnd.3 cells. Conversely, exosomes from the culture medium of HepG2 cells expressing exogenous CrebH inhibited TNFα-induced *MAdCAM-1* expression. Furthermore, exosomes isolated from *CrebH*^−/−^ mice stimulated infiltration and activation of immune cells in the liver of WT mice with IBD, while exosomes isolated from WT mice did not. These data suggest that exosomes have a protective role in IBD-induced hepatic inflammation and CrehH can regulate the contents of exosomes.

Notably, many miRNAs of exosomes isolated in plasma from *CrebH*^−/−^ mice were significantly increased compared with those of WT mice in both healthy and diseased conditions, suggesting that CrebH might regulate exosomal miRNA by unknown mechanisms. Mechanistically, exosomal miRNAs mediate post-transcriptional gene silencing by binding to the 3′-UTR or open reading frame region of the target gene [41]. Recently, Deng et al. demonstrated that miR-29a-3p could inhibit the expression of *ICAM1*, *VCAM1*, and E-selectin induced by TNFα stimulation in vitro and in vivo [34]. They also suggested that miR-29a-3p specifically suppresses TNF-R1 expression by targeting the 3′-UTR of *TNFRSF1A* [34]. Furthermore, MAdCAM-1 was induced by the TNFα signaling pathway [33]. Therefore, miR-29a-3p was selected as a potential miRNA for testing. We found that miR-29a-3p was increased in exosomes from HepG2 cells overexpressing CrebH compared with those from control cells expressing pcDNA3 and was reduced in the exosomes isolated from plasma of *CrebH*^−/−^ mice; however, the difference was not statistically significant. The miR-29a-3p mimic inhibited *MAdCAM-1* and *VAP1* expression in bEnd.3 cells. Furthermore, we found a dramatic upregulation of TNF-R1 in the livers of *CrebH*^−/−^ mice. These data suggest that miR-29a-3p might be a critical factor in regulating MAdCAM-1 expression along with another adhesion molecule such as *ICAM1*, *VCAM1*, or E-selectin. According to previous studies, miRNAs are preferentially sorted into exosomes by four potential modes, including the neural sphingomyelinase 2-associated pathway [42], heterogeneous nuclear ribonucleoprotein-associated pathway [43], 3′-end of the miRNA sequence-associated pathway, and miRNA-induced silencing complex-related pathway [44]. Thus, CrebH might be involved in these miRNA sorting systems; however, further studies are necessary to confirm this.

## Conclusion

**Fig. 8:**
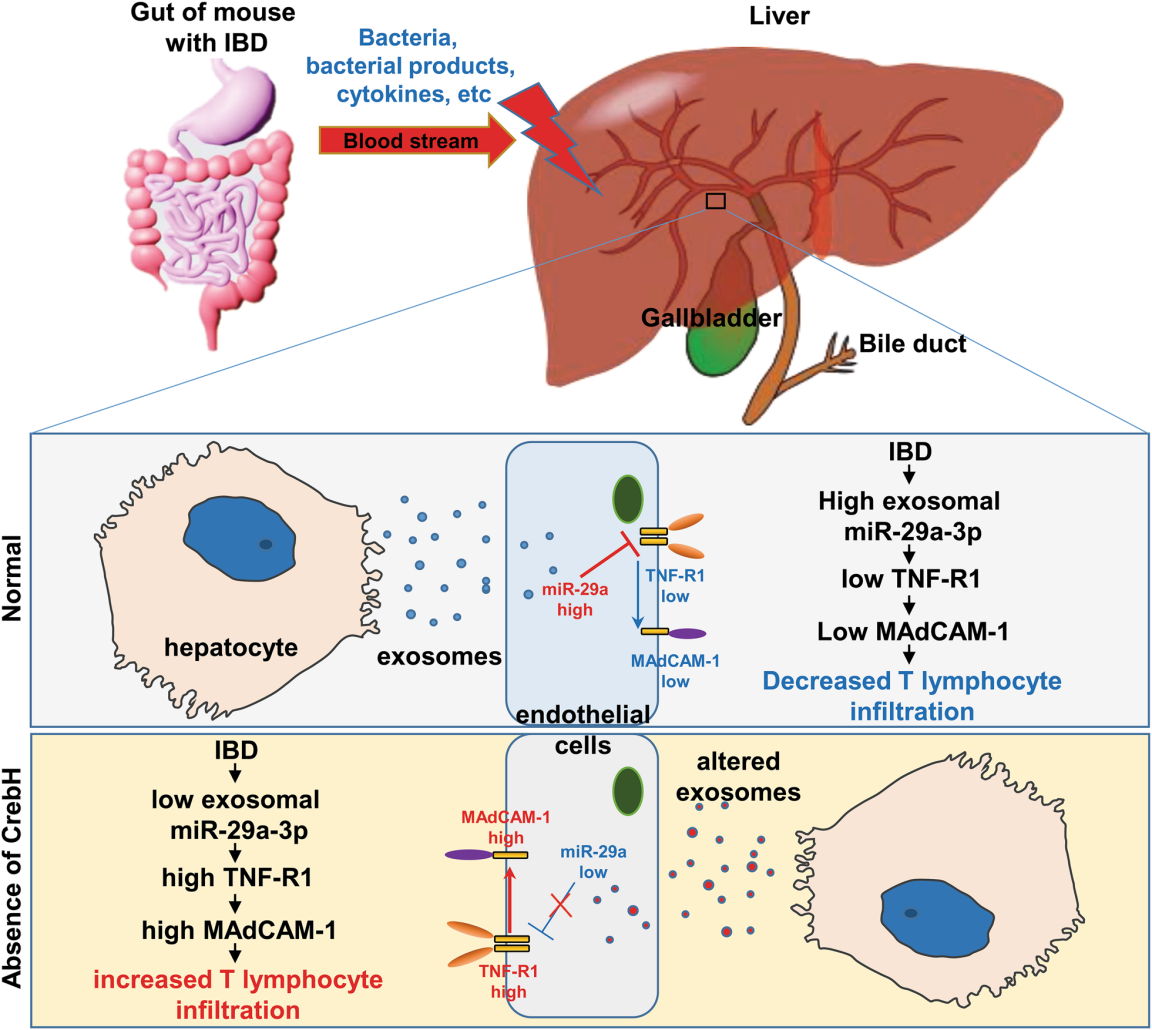
Schematic illustration of the roles of CrebH on IBD-induced liver injury. Bacteria and their products, cytokines, growth factors, and other factors move from the gut to liver through the blood stream during IBD. These contribute to liver injury via stimulation of inflammation. In normal condition, exosomal miRNA blocks the expression of MAdCAM-1 via regulation of TNF-R1. However, absence of CrebH lead to aberrant exosomal miRNA, resulting in severe liver injury

This study demonstrates that liver of *CrebH*^−/−^ mice after DSS treatment show characteristic phenotypes resembling the PSC-liver, as evidenced by increased biliary inflammation, enlarged bile ducts, upregulation of adhesion molecules such as MAdCAM-1, and high similarity of altered genes compared with PSC-liver, suggesting that *CrebH*^−/−^ mice might be a potential animal model for investigating the initial pathogenesis of liver during the progression of PSC-IBD. This study also demonstrates that exosomes play a pivotal role in protecting the subsequent pathogenesis of IBD and IBD-related liver inflammation (Fig. 8). Exosomal miRNAs are potential effector molecules, and CrebH can affect some exosomal miRNAs. Based on recent clinical studies on monoclonal antibodies against MAdCAM-1 or α4β7 [45, 46], miR-29a-3p could be an effective therapeutic strategy for IBD treatment. Therefore, this study provides new insight for future human studies.

## Supplementary Information

**Additional file1: Table S1** Primer sequences used in this study.

**Additional file 2: Figure S1 **Ablation effects of CrebH on the development of DNBS-induced IBD pathogenesis.** A** Survival of WT (n=10) and KO (n=9) mice response to DNBS. **B** Colon length of WT (n=3) and CrebH^-/-^ (n=5 ~ 6) mice treated with a vehicle or DNBS for 3 days. **C** Representative images of the colon and ileum. Bar represents 200 μm.

**Additional file 3: Figure S2 **Ablation effects of CrebH on DNBS-induced liver injury.** A** Growth images of WT and *CrebH*^*-/-*^ mice and their liver. WT and *CrebH*^*-/-*^ mice were intrarectally administrated with 3 mg of DNBS for 3 d. **B**, **C** Plasma ALT and AST levels of WT and *CrebH*^*-/-*^ mice. *P < 0.05, or ***P < 0.001. **D** Liver histology of WT and *CrebH*^*-/-*^ mice. Bar represents 200 μm.

**Additional file 4: Figure S3 **Ablation effects of CrebH in animals with PSC pathogenesis. **A** The plasma ALP levels in the plasma of WT (n = 5) and *CrebH*^*-/-*^ (n = 5) mice. *P < 0.05. **B** Representative pathological images of liver tissues stained with hematoxylin and eosin or Sirius red and Sirius red positive area (graph). The bar represents 200 μm. **C**–**F** mRNA expression of *MAdCAM-1 (C)*, *VAP1 (D)*, *ICAM1 (E)*, and *VCAM1 (F)*. *P < 0.05 or **P < 0.01. **G** Infiltration of immune cells in the liver of each group. F4/80-positive area and CD3-positive cell number were analyzed by ImageJ software. The bar represents 200 μm. *P < 0.05.

**Additional file 5: Table S2** Plasma parameters analyzed by Multiplex cytokine bead assay. Data are expressed as mean ± SEM.

**Additional file 6: Figure S4** CrebH protein expression was determined by western blotting using antibody against CrebH. HepG2 cells were transiently transfected with plasmid expressing pcDNA3, CrebH-full form, and CrebH-active form (N-terminal region) and selected by incubation with G418.

**Additional file 7: Table S3** Differently regulated miRNA lists (WC-exo vs. WD-exo).

**Additional file 8: Table S4** Differently regulated miRNA lists (WD-exo vs. KD-exo).

**Additional file 9: Table S5** Differently regulated miRNA lists (WC-exo vs. KC-exo).

## Data Availability

The RNA-seq raw data has been deposited in the Korean Nucleotide Archive (KoNA, https://kobic.re.kr/kona) under accession numbers PRJKA220165 and PRJKA220166. The datasets used and/or analysed during the current study are available from the corresponding authors on reasonable request.

## Abbreviations

ALP: Alkaline phosphatase
ALT: Alanine aminotransferase
AST: Aspartate aminotransferase
CD: Crohn’s disease
cAMP: Cyclic adenosine monophosphate
cDNA: Complementary DNA
CrebH: Cyclic adenosine monophosphate-responsive element-binding protein H
DDC: 3.5-Dieythioxycarbonyl-1,4-dihydrocollidine
DEGs: Differentially expressed genes
DNBS: Dinitrobenzene
DSS: Dextran sulfate sodium
EIMs: Extraintestinal manifestations
IBD: Inflammatory bowel disease
ICAM1: Intercellular adhesion protein 1
MAdCAM-1: Mucosal vascular addressin cell adhesion molecule 1
MF: Molecular function
MPO: Myeloperoxidase
PSC: Primary sclerosing cholangitis
TNF-R1: TNF receptor-1
UC: Ulcerative colitis
VAP1: Vascular adhesion protein 1
VCAM1: Vascular cell-adhesion molecule 1
