# Supplementary material for: CrebH protects against liver injury associated with colonic inflammation via modulation of exosomal miRNA
Source: Cell Biosci. 2023 Jun 27;13:116. doi: 10.1186/s13578-023-01065-9 (PMC10304376; doi:10.1186/s13578-023-01065-9)
Supplement: Supplementary file 1 — Additional file1: Table S1 Primer sequences used in this study. [file 13578_2023_1065_MOESM1_ESM.docx]

Table S1: Primer sequences used in this study

| **Primer lists of gene used for qRT-PCR** | | |
| --- | --- | --- |
| ***Genes*** | **Forward** | **Reverse** |
| *mIl-1β* | 5’-CTACAGGCTCCGAGATGAACAAC-3’ | 5’-TCCATTGAGGTGGAGAGCTTTC-3’ |
| *mIl-6* | 5’-TCCATCCAGTTGCCTTCTTG-3’ | 5’-TTCCACGATTTCCCAGAGAAC-3’ |
| *mTnfα* | 5’-CCCTCACACTCAGATCATCTTCT-3’ | 5’-GCTACGACGTGGGCTACAG-3’ |
| *mCrebH* | 5’-TGGTCTGGAGAACCGGATGT-3’ | 5’-GGTGTTTCAGCTGCTCCAGAA-3’ |
| *mMAdCAM-1* | 5’-CTG AGC CCT ACA TCC TGA CCT-3’ | 5’-GCT TCA CAG AGT AGC TCC CAG-3’ |
| *mVAP1* | 5’-CTT CAC CGA CTT CAT CAG CA-3’ | 5’-CCC GGA AAT AGA TGG AGT CA-3’ |
| *mICAM1* | 5’-TCACCAGGAATGTGTACCTGACA-3’ | 5’-ATCACGAGGCCCACAATGAC-3’ |
| *mVCAM1* | 5’-TGACTCCATGGCCCTCACTT-3’ | 5’-CGTCCTCACCTTCGCGTTTA-3’ |
| *mAcot3* | 5’-GAATTGGAAGTGGCCTTCTG-3’ | 5’-GTCCTTAGGGAGGTCCTCGT -3’ |
| *mCyp4a10* | 5’-TGGATTGGGTATGGTTTGCT-3’ | 5’-TCGGCCATGTTTTTCACATA-3’ |
| *mCyp4a14* | 5’-TGGGGAGATCAGATCCAAAG-3’ | 5’-CGATGCTGGAACCACTTCTT-3’ |
| *mFabp4* | 5’-TGGAAGCTTGTCTCCAGTGA-3’ | 5’-AATCCCCATTTACGCTGATG-3’ |
| *mMcam* | 5’-CAGCCAAGTGGACTGGTTTT-3’ | 5’-AAGGCGGTGCTCATATTCAC-3’ |
| *mAdamts15* | 5’-AATGGCCGCCACTACTACAG-3’ | 5’-CATCTGGCGTCAGGTGTAGA-3’ |
| *mAnxa2* | 5’-GGGGTGAAGAGGAAAGGAAC-3’ | 5’-TTGATGCTCTCCAGCATGTC-3’ |
| *mStss* | 5’-TGGGAGACATGACCAATGAA-3’ | 5’-AGCCCTTCTCTCTCCAGTCC-3’ |
| *mDcn* | 5’-AAGAAGGGGCCTTCAAGAAC-3’ | 5’-AAGCCTTTCCAACTTCACGA-3’ |
| *mItgb3* | 5’-ATCTGTCCAGCATCCAGACC-3’ | 5’-GGTTTTTGATTGCCTGTGGT-3’ |
| *mLgals1* | 5’-TCAAACCTGGGGAATGTCTC-3’ | 5’-GCGAGGATTGAAGTGTAGGC-3’ |
| *mTNF-R1* | 5’- GGCTCTGCTGATGGGGATAC-3’ | 5’- ACTTGGTGCAGCAGATGGAA-3’ |
| *18s* | 5’-GACACGGACAGGATTGACAGATTGATAG-3’ | 5’-GACACGGACAGGATTGACAGATTGATAG-3’ |
| **Primer lists of mouse miRNA used for RT and qRT-PCR** | | |
| **miRNAs** | **Sequences** | |
| miR-29a-3p | **RT**: 5’- GTCGTATCCAGTGCAGGGTCCGAGGTATTCGCACTGGATACG  ATAACCG -3’  **Forward**:5’- CGTAGCACCATCTGAAATCG -3’ | |
| U6 | **RT**: 5’-GTCGTATCCAGTGCAGGGTCCGAGGTATTCGCACTGGATACG  ACAAAATG-3’  **Forward**:5’-GCGCGTCGTGAAGCGTTC-3’ | |
|  | **Universal Reverse**: 5’-GTGCAGGGTCCGAGGT-3’ | |
|  | **mimic of miR-29a**: 5’-ACUGAUUUCUUUUGGUGUUCAG-3’ | |
