# Supplementary figures and images for: CrebH protects against liver injury associated with colonic inflammation via modulation of exosomal miRNA
Source: Cell Biosci. 2023 Jun 27;13:116. doi: 10.1186/s13578-023-01065-9 (PMC10304376; doi:10.1186/s13578-023-01065-9)

## Slide 1
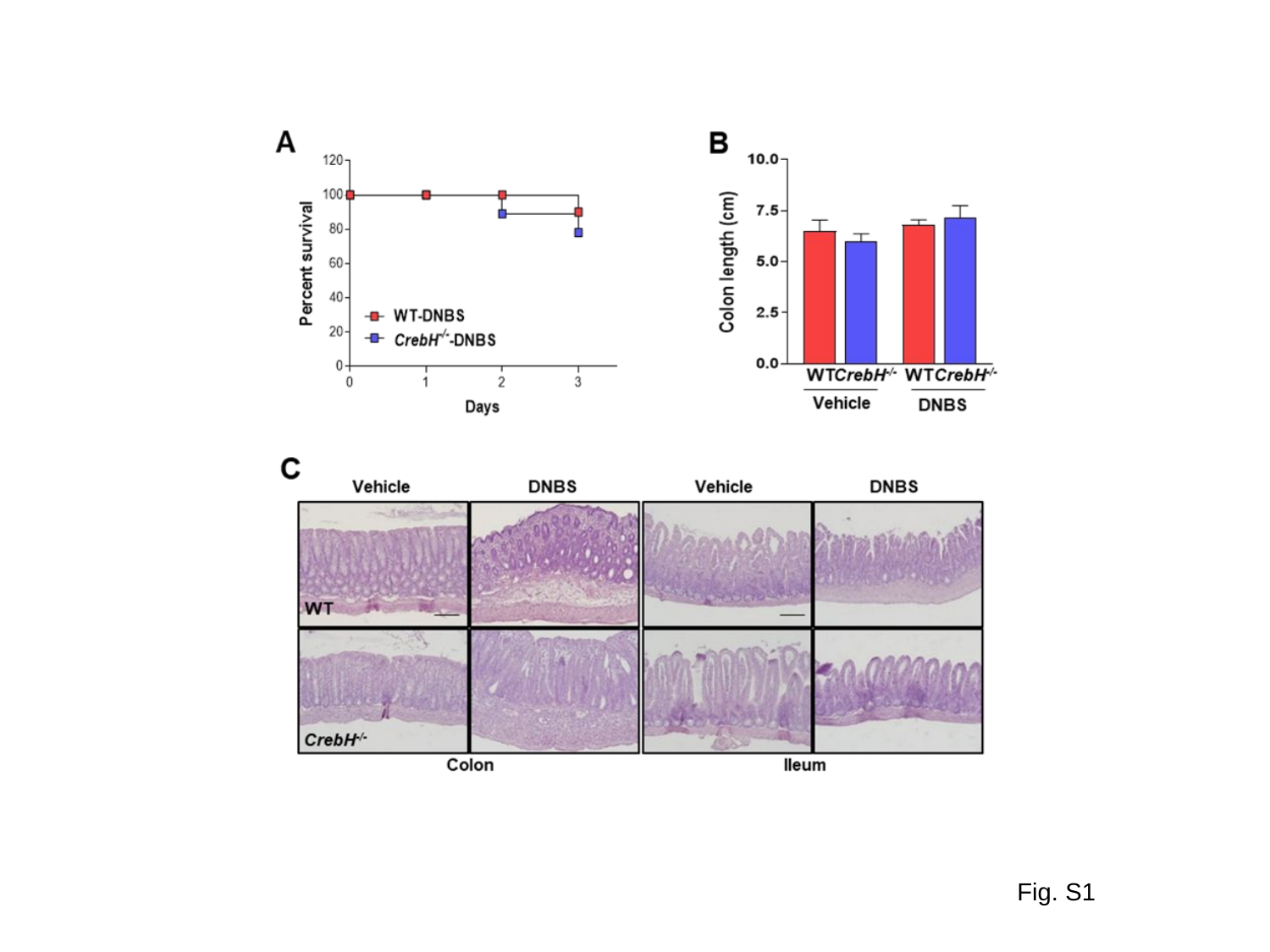

Fig. S1

Supplement: Supplementary file 2 — Additional file 2: Figure S1 Ablation effects of CrebH on the development of DNBS-induced IBD pathogenesis. A Survival of WT (n=10) and KO (n=9) mice response to DNBS. B Colon length of WT (n=3) and CrebH-/- (n=5 ~ 6) mice treated with a vehicle or DNBS for 3 days. C Representative images of the colon and ileum. Bar represents 200 μm. [file 13578_2023_1065_MOESM2_ESM.pptx]

## Slide 1
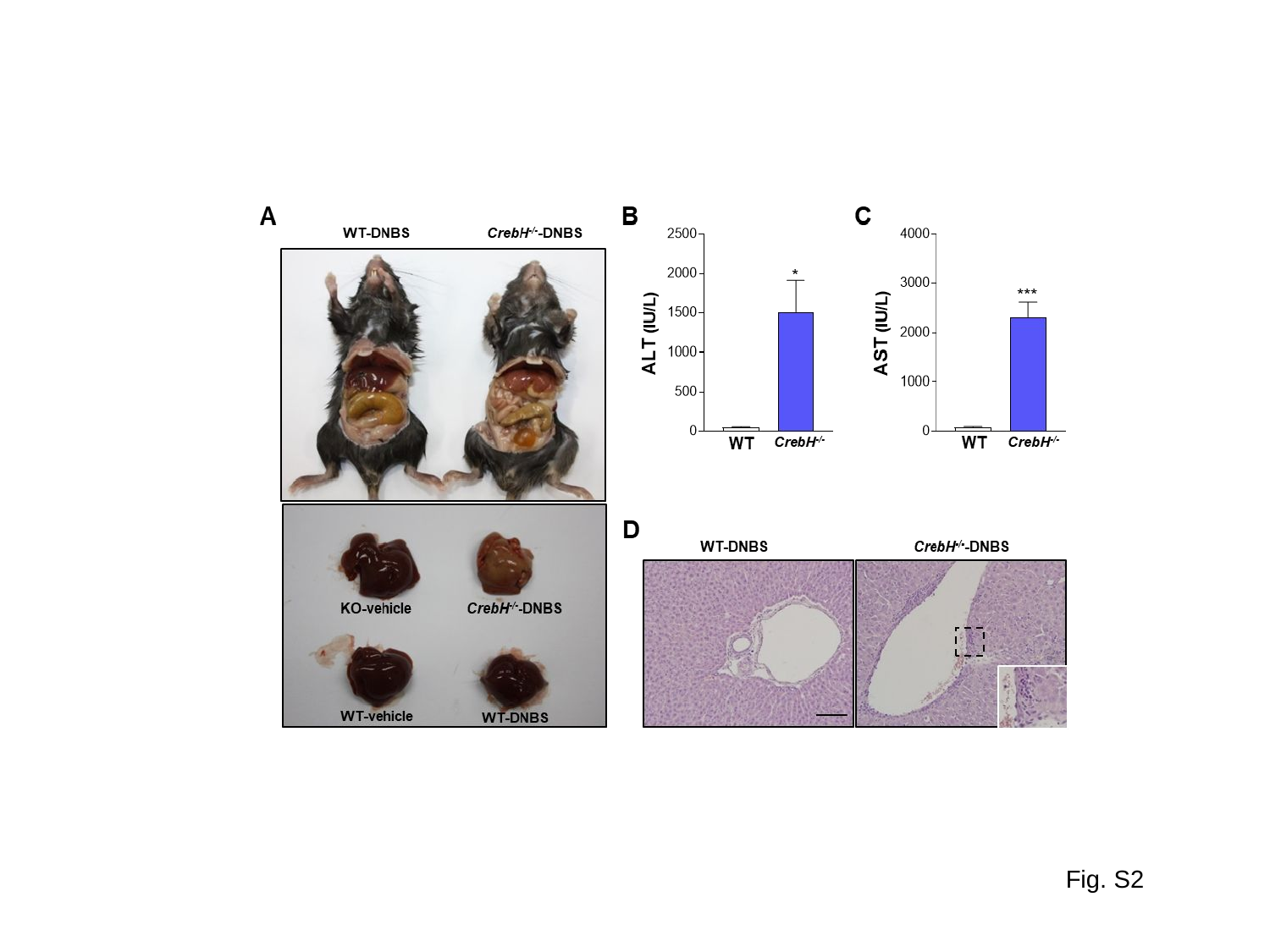

Fig. S2

Supplement: Supplementary file 3 — Additional file 3: Figure S2 Ablation effects of CrebH on DNBS-induced liver injury. A Growth images of WT and CrebH-/- mice and their liver. WT and CrebH-/- mice were intrarectally administrated with 3 mg of DNBS for 3 d. B, C Plasma ALT and AST levels of WT and CrebH-/- mice. *P < 0.05, or ***P < 0.001. D Liver histology of WT and CrebH-/- mice. Bar represents 200 μm. [file 13578_2023_1065_MOESM3_ESM.pptx]

## Slide 1
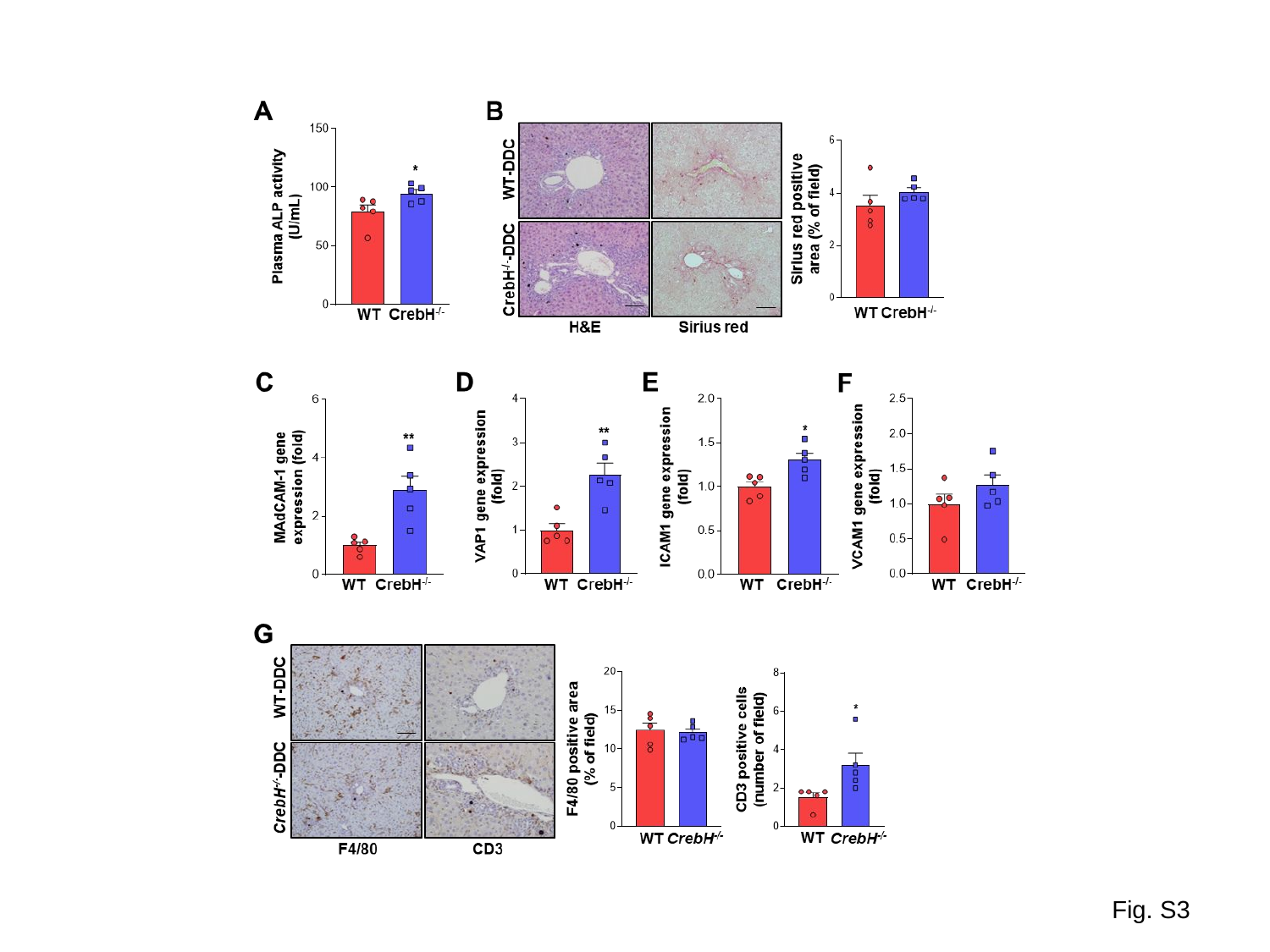

Fig. S3

Supplement: Supplementary file 4 — Additional file 4: Figure S3 Ablation effects of CrebH in animals with PSC pathogenesis. A The plasma ALP levels in the plasma of WT (n = 5) and CrebH-/- (n = 5) mice. *P < 0.05. B Representative pathological images of liver tissues stained with hematoxylin and eosin or Sirius red and Sirius red positive area (graph). The bar represents 200 μm. C–F mRNA expression of MAdCAM-1 (C), VAP1 (D), ICAM1 (E), and VCAM1 (F). *P < 0.05 or **P < 0.01. G Infiltration of immune cells in the liver of each group. F4/80-positive area and CD3-positive cell number were analyzed by ImageJ software. The bar represents 200 μm. *P < 0.05. [file 13578_2023_1065_MOESM4_ESM.pptx]

## Slide 1
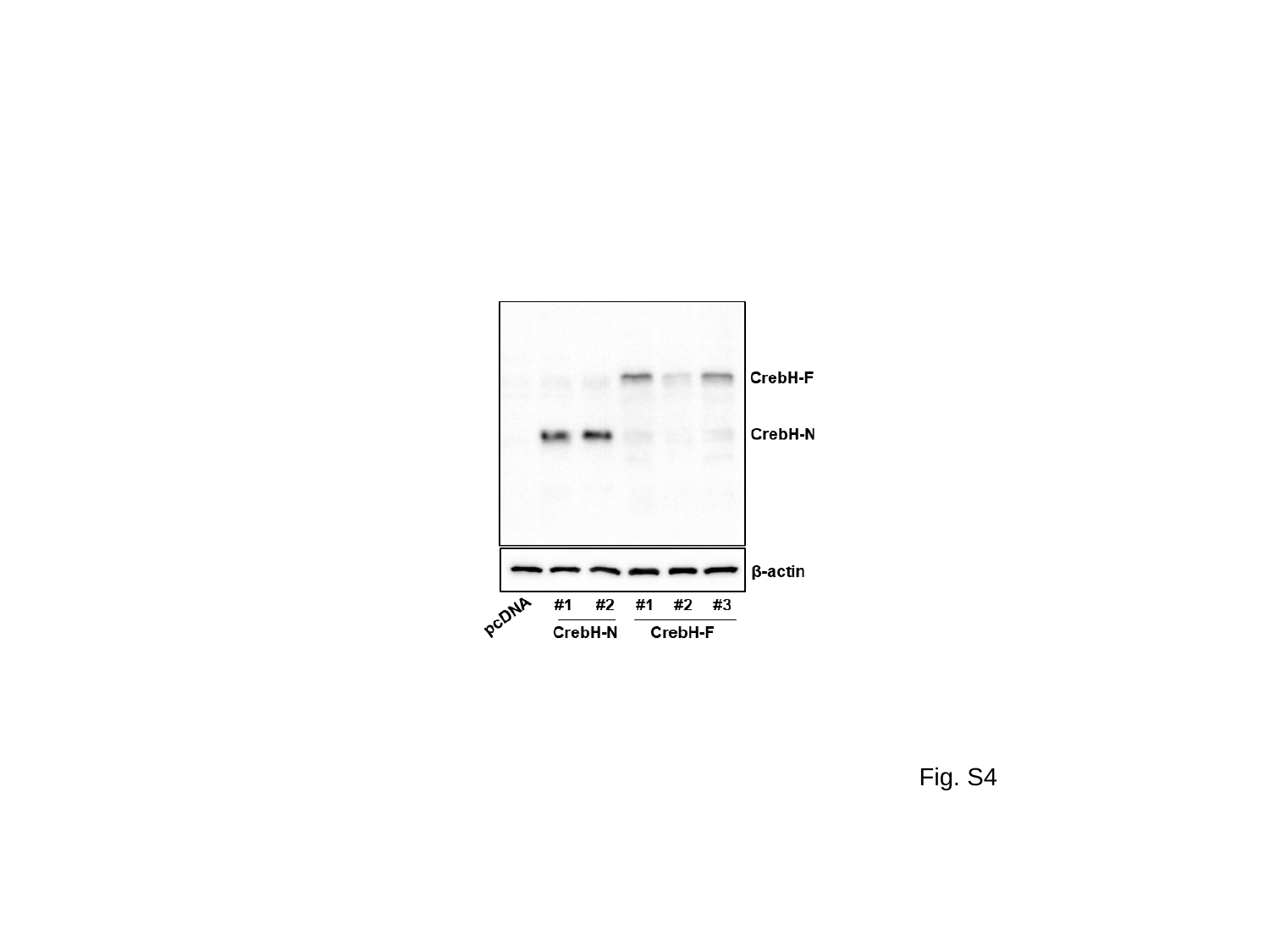

Fig. S4

Supplement: Supplementary file 6 — Additional file 6: Figure S4 CrebH protein expression was determined by western blotting using antibody against CrebH. HepG2 cells were transiently transfected with plasmid expressing pcDNA3, CrebH-full form, and CrebH-active form (N-terminal region) and selected by incubation with G418. [file 13578_2023_1065_MOESM6_ESM.pptx]
