# Supplementary material for: CrebH protects against liver injury associated with colonic inflammation via modulation of exosomal miRNA
Source: Cell Biosci. 2023 Jun 27;13:116. doi: 10.1186/s13578-023-01065-9 (PMC10304376; doi:10.1186/s13578-023-01065-9)
Supplement: Supplementary file 5 — Additional file 5: Table S2 Plasma parameters analyzed by Multiplex cytokine bead assay. Data are expressed as mean ± SEM. [file 13578_2023_1065_MOESM5_ESM.docx]

Table S2. Plasma parameters analyzed by Multiplex cytokine bead assay. Data are expressed as mean ± SEM.

| **Names** | **WT**  **(n=8)** | **CrebH**^-/-^  **(n=8)** | **p-value** |
| --- | --- | --- | --- |
| IL-1α | 6.11 ± 4.75 | 5.51 ± 2.82 | 0.78 |
| IL-1β | 9.54 ± 8.21 | 6.93 ± 3.38 | 0.45 |
| IL-2 | 22.1 ± 10.91 | 26.45 ± 5.2 | 0.36 |
| IL-3 | 5.61 ± 4.78 | 5.88 ± 1.67 | 0.89 |
| IL-4 | 8.33 ± 6.33 | 8.13 ± 2.55 | 0.94 |
| IL-5 | 18.4 ± 13.95 | 24.61 ± 8.26 | 0.33 |
| IL-6 | 9.4 ± 6.19 | 11.1 ± 3.37 | 0.53 |
| IL-9 | 14.94 ± 15.79 | 17.41 ± 8.63 | 0.72 |
| IL-10 | 32.79 ± 24.64 | 26.75 ± 14.31 | 0.58 |
| IL-12p40 | 120.41 ± 57.88 | 163.67 ± 46.31 | 0.14 |
| IL-12p70 | 98.58 ± 77.43 | 102.75 ± 49.23 | 0.91 |
| IL-13 | 19.66 ±17.08 | 34.9 ± 48.2 | 0.44 |
| IL-17a | 10.25 ± 6.94 | 11.95 ± 4.0 | 0.58 |
| TNFα | 101.11 ± 72.4 | 128.57 ± 47.75 | 0.42 |
| IFN-γ | 28.72 ± 20.13 | 33.08 ± 10.32 | 0.62 |
| KC | 61.09 ± 34.49 | 75.46 ± 54.73 | 0.57 |
| MCP-1 | 227.79 ± 109.92 | 215.25 ± 80.01 | 0.81 |
| MIP-1α | 2.92 ± 2.37 | 3.48 ± 1.54 | 0.61 |
| MIP-1β | 48.81 ± 23.67 | 56.87 ± 12.27 | 0.44 |
| MIP-2 | 2.93 ± 1.65 | 2.9 ± 0.76 | 0.96 |
| RANTES | 18.37 ± 21.43 | 22.93 ± 16.22 | 0.66 |
| Eotaxin | 239.89 ± 155.75 | 359.46 ± 101.28 | 0.12 |
| GM-SCF | 37.1 ± 16.19 | 44.29 ± 7.12 | 0.30 |
| G-CSF | 157.61 ± 160.56 | 165.8 ± 65.43 | 0.90 |
