# Supplementary material for: CrebH protects against liver injury associated with colonic inflammation via modulation of exosomal miRNA
Source: Cell Biosci. 2023 Jun 27;13:116. doi: 10.1186/s13578-023-01065-9 (PMC10304376; doi:10.1186/s13578-023-01065-9)
Supplement: Supplementary file 7 — Additional file 7: Table S3 Differently regulated miRNA lists (WC-exo vs. WD-exo). [file 13578_2023_1065_MOESM7_ESM.docx]

Table S3: Differently regulated miRNA lists (WC-exo vs. WD-exo).

| **Probe name** | **Fold** | ***p* value** | **Probe name** | **Fold** | ***p* value** |
| --- | --- | --- | --- | --- | --- |
| mmu-miR-1196 | 48.13 | 0.038 | mmu-miR-208a | -3.56 | 0.046 |
| mmu-miR-2134 | 14.72 | 0.041 | mmu-miR-330 | -3.57 | 0.047 |
| mmu-miR-1944 | 7.64 | 0.017 | mmu-miR-103 | -3.7 | 0.025 |
| mmu-miR-709 | 5.21 | 0.026 | mcmv-miR-M23-1-5p | -3.76 | 0.009 |
| mcmv-miR-2135 | 3.6 | 0.041 | mmu-miR-324-5p | -3.76 | 0.016 |
| mmu-miR-1224 | 3.08 | 0.009 | mmu-miR-1195 | -3.85 | 0.024 |
| mmu-miR-29a | 2.26 | 0.003 | mmu-miR-872 | -3.87 | 0.017 |
| mmu-miR-22 | 2.15 | 0.039 | mmu-miR-541 | -3.92 | 0.045 |
| mmu-miR-205 | -2 | 0.035 | mmu-miR-188-5p | -3.93 | 0.033 |
| mmu-miR-15a | -2.05 | 0.023 | mmu-miR-698 | -4 | 0.045 |
| mmu-miR-691 | -2.1 | 0.037 | mmu-miR-144 | -4.13 | 0.038 |
| mmu-miR-let-7g | -2.21 | 0.038 | mmu-miR-770-3p | -4.13 | 0.026 |
| mmu-miR-125b-3p | -2.27 | 0.048 | mmu-miR-432 | -4.24 | 0.029 |
| mmu-miR-339-3p | -2.47 | 0.019 | mmu-miR-883b-5p | -4.29 | 0.01 |
| mmu-miR-30e | -2.51 | 0.017 | mmu-miR-497 | -4.4 | 0.019 |
| mmu-miR-491 | -2.58 | 0.026 | mmu-miR-880 | -4.4 | 0.012 |
| mmu-miR-125-5p | -2.67 | 0.011 | mmu-miR-760 | -4.49 | 0.007 |
| mmu-miR-138 | -2.79 | 0.011 | mmu-miR-135b | -4.64 | 0.021 |
| mmu-miR-1941-3p | -2.83 | 0.039 | mmu-miR-324-3p | -4.74 | 0.032 |
| mcmv-miR-m21-1 | -2.84 | 0.05 | mmu-miR-369-5p | -4.74 | 0.005 |
| mghv-miR-M1-7-3p | -2.84 | 0.019 | mmu-miR-351 | -4.85 | 0.03 |
| mmu-miR-675-5p | -2.84 | 0.02 | mmu-miR-1927 | -4.85 | 0.03 |
| mmu-miR-883a-3p | -2.94 | 0.013 | mmu-miR-743b-5 | -4.85 | 0.015 |
| mmu-miR-322 | -3.02 | 0.05 | mmu-miR-463 | -5.15 | 0.007 |
| mmu-miR-1190 | -3.04 | 0.002 | mmu-miR-105 | -5.2 | 0.02 |
| mmu-miR-693-5p | -3.06 | 0.016 | mmu-miR-216b | -5.34 | 0.005 |
| mmu-miR-2139 | -3.11 | 0.038 | mmu-miR-1894-3p | -5.51 | 0.02 |
| mcmv-miR-m22-1 | -3.18 | 0.022 | mmu-miR-882 | -5.77 | 0.015 |
| mmu-miR-3072 | -3.18 | 0.029 | mmu-miR-34b-5p | -5.88 | 0.043 |
| mghv-miR-M1-1 | -3.23 | 0.002 | mmu-miR-m108-2-5p.1 | -5.95 | 0.003 |
| mmu-miR-340-3p | -3.31 | 0.023 | mcmv-miR-411 | -6.05 | 0.028 |
| mmu-miR-1190 | -3.48 | 0.043 | mmu-miR-666-5p | -6.47 | 0.03 |
| mmu-miR-590-3p | -3.49 | 0.042 | mmu-miR-3099 | -6.79 | 0.029 |
