# Supplementary material for: CrebH protects against liver injury associated with colonic inflammation via modulation of exosomal miRNA
Source: Cell Biosci. 2023 Jun 27;13:116. doi: 10.1186/s13578-023-01065-9 (PMC10304376; doi:10.1186/s13578-023-01065-9)
Supplement: Supplementary file 8 — Additional file 8: Table S4 Differently regulated miRNA lists (WD-exo vs. KD-exo). [file 13578_2023_1065_MOESM8_ESM.docx]

Table S4: Differently regulated miRNA lists (WD-exo vs. KD-exo).

| **Probe name** | **Fold** | ***p* value** | **Probe name** | **Fold** | ***p* value** |
| --- | --- | --- | --- | --- | --- |
| mmu-miR-1988 | 9.35 | 0.01 | mmu-miR-463 | 3.73 | 0.009 |
| mmu-miR-135b | 8.17 | 0.01 | mmu-miR-26a | 3.72 | 0.02 |
| mmu-miR-32 | 8.14 | 0.011 | mmu-miR-504 | 3.72 | 0.018 |
| mmu-miR-105 | 7.73 | 0.008 | mmu-miR-760 | 3.72 | 0.011 |
| mmu-miR-880 | 7.3 | 0.028 | mmu-miR-201 | 3.7 | 0.006 |
| mcmv-miR-m01-2 | 6.73 | 0.039 | mmu-miR-654-3p | 3.65 | 0.025 |
| mcmv-m108-2-5p.1 | 6.35 | 0.012 | mmu-miR-879 | 3.51 | 0.016 |
| mmu-miR-351 | 5.92 | 0.024 | mmu-miR-1901 | 3.38 | 0.043 |
| mmu-miR-485 | 5.87 | 0.049 | mmu-miR-495 | 3.38 | 0.017 |
| mcmv-miR-m108-1 | 5.6 | 0.05 | mmu-miR-1934 | 3.26 | 0.027 |
| mmu-1894-5p | 5.27 | 0.021 | mmu-miR-491 | 3.26 | 0.017 |
| mcmv-miR-M23-1-5p | 5.06 | 0.046 | mmu-miR-466a/466b | 3.23 | 0.014 |
| mmu-miR-339-3p | 5.04 | 0.006 | mmu-miR-494 | 3.15 | 0.045 |
| mmu-miR-684 | 4.96 | 0.007 | mmu-miR-770-3p | 3.13 | 0.047 |
| mmu-miR-373-5p | 4.95 | 0.01 | mmu-miR-675-5p | 3.03 | 0.026 |
| mmu-miR-683 | 4.89 | 0.014 | mmu-miR-96 | 2.97 | 0.048 |
| mmu-miR-666-5p | 4.77 | 0.05 | mmu-miR-369-5p | 2.96 | 0.047 |
| mmu-miR-702 | 4.75 | 0.028 | mmu-miR-297b-5p | 2.96 | 0.041 |
| mmu-miR-599 | 4.6 | 0.012 | mmu-miR-1194 | 2.95 | 0.043 |
| mmu-miR-1954 | 4.6 | 0.01 | mmu-miR-138 | 2.81 | 0.028 |
| mmu-miR-1952 | 4.58 | 0.035 | mmu-miR-669j | 2.76 | 0.023 |
| mmu-miR-217 | 4.5 | 0.041 | mmu-miR-666-3p | 2.75 | 0.022 |
| mmu-miR-409-3p | 4.37 | 0.01 | mmu-miR-1186b | 2.72 | 0.035 |
| mmu-miR-693-5p | 4.27 | 0.043 | mmu-miR-469 | 2.69 | 0.033 |
| mmu-miR-542-5p | 4.26 | 0.022 | mmu-miR-1191 | 2.63 | 0.021 |
| mmu-miR-1930 | 4.25 | 0.027 | mmu-miR-30e | 2.62 | 0.032 |
| mmu-miR-346 | 4.2 | 0.01 | mmu-miR-466i | 2.55 | 0.009 |
| mmu-miR-188-5p | 4.11 | 0.032 | mmu-miR-433 | 2.52 | 0.015 |
| mmu-miR-330 | 3.97 | 0.008 | mmu-miR-340-5p | 2.5 | 0.043 |
| mmu-miR-1941-3p | 3.95 | 0.029 | mmu-miR-1905 | 2.35 | 0.044 |
| mmu-miR-1968 | 3.81 | 0.039 | mmu-miR-1941-5p | 2.14 | 0.033 |
| mmu-miR-453 | 3.81 | 0.038 | mmu-miR-19a | -3.69 | 0.041 |
