# Supplementary material for: CrebH protects against liver injury associated with colonic inflammation via modulation of exosomal miRNA
Source: Cell Biosci. 2023 Jun 27;13:116. doi: 10.1186/s13578-023-01065-9 (PMC10304376; doi:10.1186/s13578-023-01065-9)
Supplement: Supplementary file 9 — Additional file 9: Table S5 Differently regulated miRNA lists (WC-exo vs. KC-exo). [file 13578_2023_1065_MOESM9_ESM.docx]

Table S5: Differently regulated miRNA lists (WC-exo vs. KC-exo).

| **Probe name** | **Fold** | ***p* value** | **Probe name** | **Fold** | ***p* value** |
| --- | --- | --- | --- | --- | --- |
| mmu-miR-376a | 6.67 | 0.038 | mmu-miR-539 | 2.73 | 0.029 |
| mmu-miR-712 | 4.89 | 0.013 | mmu-miR-434-5p | 2.65 | 0.045 |
| mmu-miR-183 | 4.76 | 0.036 | mmu-miR-744 | 2.64 | 0.048 |
| mmu-miR-1906 | 3.64 | 0.012 | mmu-miR-1193 | 2.51 | 0.007 |
| mmu-miR-346 | 3.58 | 0.036 | mmu-miR-295 | 2.47 | 0.011 |
| mmu-miR-688 | 3.5 | 0.031 | mghv-miR-M1-6 | 2.41 | 0.007 |
| mmu-miR-2135 | 3.34 | 0.044 | mmu-miR-297b-3p | 2.41 | 0.043 |
| mmu-miR-302a | 3.34 | 0.024 | mmu-miR-128 | 2.41 | 0.031 |
| mmu-miR-2145 | 3.27 | 0.024 | mmu-miR-1963 | 2.41 | 0.018 |
| mghv-miR-M1-9 | 3.26 | 0.038 | mghv-miR-M1-8 | 2.39 | 0.02 |
| mmu-miR-469 | 3.23 | 0.044 | mmu-miR-210 | 2.37 | 0.028 |
| mmu-miR-151-5p | 3.04 | 0.003 | mmu-miR-132 | 2.3 | 0.024 |
| mmu-miR-380-5p | 3.04 | 0.037 | mmu-miR-883a-3p | 2.27 | 0.022 |
| mmu-miR-1948 | 2.92 | 0.025 | mmu-miR-675-5p | 2.11 | 0.015 |
| mmu-miR-1930 | 2.91 | 0.041 | mmu-miR-150 | -2.06 | 0.021 |
| mmu-miR-582-5p | 2.86 | 0.035 | mmu-miR-324-3p | -2.08 | 0.026 |
| mmu-miR-470 | 2.8 | 0.027 | mmu-miR-423-5p | -2.14 | 0.049 |
| mmu-miR-669h-5p | 2.8 | 0.001 | mmu-miR-150 | -2.54 | 0.03 |
| mmu-miR-137 | 2.76 | 0.027 |  |  |  |
